# Conservation and evolutionary divergence in the activity of receptor-regulated smads

**DOI:** 10.1186/2041-9139-3-22

**Published:** 2012-10-01

**Authors:** Gina M Sorrentino, William Q Gillis, Jamina Oomen-Hajagos, Gerald H Thomsen

**Affiliations:** 1 Department of Anatomical Sciences, Stony Brook University, Health Science Center T-8, Stony Brook, NY, 11794-8018, USA; 2 Department of Biochemistry and Cell Biology, Stony Brook University, Life Sciences Building room 450, Stony Brook, NY, 11794-5215, USA; 3 Graduate Program in Genetics, Stony Book University, Life Sciences Building room 120, Stony Brook, NY, USA

**Keywords:** TGFβ, BMP, Smad, Animal body patterning, Evolution of signal transduction

## Abstract

**Background:**

Activity of the Transforming growth factor-β (TGFβ) pathway is essential to the establishment of body axes and tissue differentiation in bilaterians. Orthologs for core pathway members have been found in all metazoans, but uncertain homology of the body axes and tissues patterned by these signals raises questions about the activities of these molecules across the metazoan tree. We focus on the principal canonical transduction proteins (R-Smads) of the TGFβ pathway, which instruct both axial patterning and tissue differentiation in the developing embryo. We compare the activity of R-Smads from a cnidarian (*Nematostella vectensis*), an arthropod (*Drosophila melanogaster*), and a vertebrate (*Xenopus laevis*) in *Xenopus* embryonic assays.

**Results:**

Overexpressing NvSmad1/5 ventralized *Xenopus* embryos when expressed in dorsal blastomeres, similar to the effects of *Xenopus* Smad1. However, NvSmad1/5 was less potent than XSmad1 in its ability to activate downstream target genes in *Xenopus* animal cap assays. NvSmad2/3 strongly induced general mesendodermal marker genes, but weakly induced ones involved in specifying the Spemann organizer. NvSmad2/3 was unable to induce a secondary trunk axis in *Xenopus* embryos, whereas the orthologs from *Xenopus* (XSmad2 and XSmad3) and *Drosophila* (dSmad2) were capable of doing so. Replacement of the NvSmad2/3 MH2 domain with the *Xenopus* XSmad2 MH2 slightly increased its inductive capability, but did not confer an ability to generate a secondary body axis.

**Conclusions:**

Vertebrate and cnidarian Smad1/5 have similar axial patterning and induction activities, although NvSmad1/5 is less efficient than the vertebrate gene. We conclude that the activities of Smad1/5 orthologs have been largely conserved across Metazoa. NvSmad2/3 efficiently activates general mesendoderm markers, but is unable to induce vertebrate organizer-specific genes or to produce a secondary body axis in *Xenopus*. Orthologs dSmad2 and XSmad3 generate a secondary body axis, but activate only low expression of organizer-specific genes that are strongly induced by XSmad2. We suggest that in the vertebrate lineage, Smad2 has evolved a specialized role in the induction of the embryonic organizer. Given the high level of sequence identity between Smad orthologs, this work underscores the functional importance of the emergence and fixation of a few divergent amino acids among orthologs during evolution.

## Background

In developing animal embryos the Transforming Growth Factor-β (TGFβ) superfamily of ligands and signaling pathways regulate cell fate decisions, pattern formation, growth and organogenesis. Canonical TGFβ signals are transduced by Smad proteins operating in either of two major signaling branches, the bone morphogenetic protein (BMP) and Activin/Nodal pathways. The unique receptor-regulated Smad (R-Smad) protein sequences determine the specificity of each R-Smad for upstream receptors and downstream cofactors and target genes. Recently, orthologs of the core members of the TGFβ pathway have been identified outside of Bilateria, in animals that lack the degree of complexity seen in bilaterian symmetry and tissue-types [1]. These animals possess TGFβ genes even though none have a true dorsoventral axis or mesoderm, and the sponge lacks definitive germ layers altogether. TGFβ superfamily ligands and their signal transduction components are not found in the choanoflagellate *Monosiga brevicollis* (the eukaryotic outgroup to Metazoa), which indicates that this growth factor system is restricted to Metazoa [1-3].

Discovery of key conserved developmental gene pathways has led to the paradigm of a shared ‘genetic toolkit’: a gene network that generates the variety of animal body forms by differential deployment. Work has been done to reveal the evolutionary history of many gene networks by mapping their presence or absence onto phylogenetic trees. It has been tempting to reconstruct the presence of morphological features along with the presence of a gene network in animal ancestors at key nodes, such as the ancestors of Bilateria and Eumetazoa [4]. However, some authors reject these reconstructions on the grounds that conservation of genes involved in core genetic regulatory networks does not necessitate the presence of the particular morphologies known to be regulated by these networks [5]. These disagreements highlight the need for functional testing when studying the meaning of these orthologous gene networks.

We approached the question of functional conservation by testing the ability of non-bilaterian gene products to function in a developing vertebrate *in vivo*. We focus on the Smad proteins, which operate both as intracellular transducers of TGFβ family receptor signals and as transcription factors. Failure of Smad signaling and abnormal downstream gene regulation causes fundamental disruption of body axes and cell fate determination. Three subtypes of Smads are involved in TGFβ signaling [6-8], the receptor-regulated (R), the common (Co) and the inhibitory Smads (I). R-Smads are phosphorylated at a C-terminal pair of serine residues when an extracellular ligand binds to Type I and II receptors, forming a signaling complex. Phosphorylated R-Smads then bind to a Co-Smad to form a trimeric complex that facilitates additional interactions with transcription factors on promoter elements of target genes. Smad signaling is regulated at the level of receptors and R-Smad/Co-Smad complexes by I-Smads [6]. With a few exceptions, most non-vertebrate taxa have four Smad genes, an R-Smad in the Activin/Nodal pathway (AR-Smad), an R-Smad in the BMP pathway (BR-Smad), a Co-Smad, and an I-Smad. Vertebrates typically have multiple copies of each due to gene duplication events [3], which raise major questions about whether duplicated Smads have retained ancestral activities and/or evolved divergent functions.

**Figure 1:**
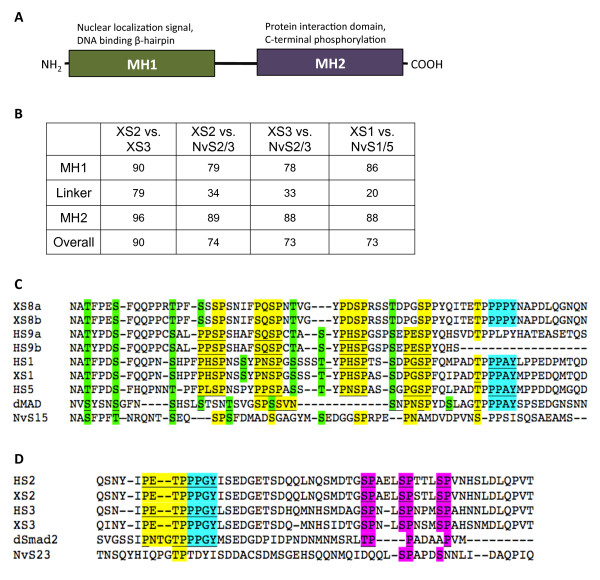
**R-Smads are defined by two conserved protein domains.** (**A**) Diagram of a typical R-Smad showing the two conserved domains (MH1 and MH2) with important regions noted. (**B**) A table of pairwise percent identity of the MAD homology domains and the non-conserved linker regions of *Xenopus* and *Nematostella* R-Smads [see Additional file 1 for a detailed amino acid alignment and Additional file 2 for a table of GenBank accession numbers]. (**C**) Alignment of relevant sections of the Smad1/5/8 linker regions from multiple taxa. The Smurf1 site PPXY motif is highlighted in cyan, MAPK recognition sites are highlighted in yellow, and GSK3 consensus sites are highlighted in green. (**D**) Alignment of relevant sections of the Smad2/3 linker regions from multiple taxa. The Smurf2 site PPXY motif is highlighted in cyan, MAPK recognition sites are highlighted in yellow, and proline-directed kinase sites are highlighted in magenta. Underlined sequences in (**C**) and (**D**) indicate consensus sites identified for the particular protein and species in the literature; all other highlights reflect our inferences based on the alignments.

Smads are considered highly conserved in their biochemical and biological functions, and they are structurally defined by the presence of two characteristic ‘MAD homology’ (MH) domains that determine functionality. Generally speaking, the N-terminal MH1 domain binds directly to DNA and contains a nuclear localization signal, and the C-terminal MH2 domain contains binding sites for the numerous potential protein co-factors that make up the transcriptional complexes (Figure 1A) [6,8]. R-Smad proteins terminate at a consensus SSXS polypeptide, of which the last two serines become phosphorylated in response to receptor activation [6] (see Additional file 1). Co- and I-Smads lack this consensus. The MH1 and MH2 domains are separated by a linker region that can be highly variable among species and even Smad subtypes, but serves important regulatory functions by providing sites for non-TGFβ receptor-driven phosphorylation and targeting by E3 ubiquitin ligases [8].

Vertebrates have three BR-Smads that transduce BMP signals - Smad1, Smad5, and Smad8/9 (see Additional file 1). In *Xenopus*, XSmad1 is the major embryonic intracellular transducer of BMP signals, and its ectopic expression in dorsal embryonic regions mimics the effects of BMP overexpression such as loss of dorsal cell identity resulting in tadpoles that are almost entirely composed of ventral tissues, lacking heads and neural tissues as a consequence of respecification [9]. Functional conservation of BR-Smad orthologs across taxa has been shown by the ectopic expression of dMad, the XSmad1 ortholog from *Drosophila*, that when injected dorsally into *Xenopus* embryos causes the same catastrophic loss of head and neural tissues as overexpression of the native XSmad1 [9].

*Xenopus laevis*, like most vertebrates, has two AR-Smads in the Activin/Nodal pathway - Smad2 and Smad3. Overexpression of XSmad2 induces dorsal mesoderm in pluripotent *Xenopus* animal caps [10] and a secondary body (trunk) axis in whole *Xenopus* embryos [11]. A dominant negative form of XSmad2 inhibits anterior mesoderm development and decreases induction of organizer genes such as *chordin, goosecoid*, and *cerberus*[12]. Less is known about the specific function of XSmad3, but evidence suggests functional specialization of Smad2 and Smad3 [13]. In *Xenopus*, XSmad2 is present maternally and throughout gastrulation, neurulation and tadpole stages and is significantly more abundant than XSmad3, which is present as low abundance maternal RNA that disappears in early gastrulation and reappears in tailbud tadpoles in specialized tissues [14]. The potential for these genes to have discrete functions is even more pronounced in the mouse. *Smad2* knockout mice fail to gastrulate and exhibit early embryonic lethality, whereas *Smad3* knockouts are born alive but die within 1 to 10 months due to cancer and immune deficiencies [15]. Zebrafish have three copies of the AR-Smads - *Smad2, Smad3a,* and *Smad3b*[16]. Reports on their function and relative developmental importance are conflicting, but they appear to be distinct as well [16-18]. However, whether this distinction is based on regulatory sequences or primary protein sequence is unclear.

In contrast to vertebrates, most non-vertebrate animals have just two R-Smads. With respect to the Activin-like pathway in *Drosophila*, an AR-Smad called *dSmad2* has been described but its activity and significance appears to be quite different than Smad2/3 in vertebrates [19,20]. The protein dSmad2 is activated by the Activin-type receptor Baboon, and loss of Baboon function (and thus dSmad2 function) causes minor problems with cell proliferation and growth, but does not affect body patterning [20]. In fact, dSmad2 overexpression in prospective ectoderm of *Xenopus* animal caps causes Activin-like induction of mesoderm [19], but the level to which dSmad2 shares functional homology with vertebrate Smad2 or Smad3 was not tested.

Smad family members have been identified in all metazoan clades, but the extent to which there is (or is not) functional conservation among the Smads, particularly across highly divergent taxa such as non-bilaterians and chordates, is an important question to answer that will inform the evolution of this protein family. In the present study, we used qualitative and quantitative methods to examine whether the functions of the R-Smads have been conserved sufficiently during metazoan evolution to allow R-Smads from a cnidarian to participate in the TGFβ signal transduction network during early vertebrate embryogenesis. We have chosen two exemplar taxa for this study, *Xenopus laevis* (the African clawed frog, a model organism for functional studies) and the model cnidarian *Nematostella vectensis* (the starlet sea anemone). The *Nematostella* BR-Smad ortholog, NvSmad1/5, has been identified, and a *Nematostella* AR-Smad ortholog (NvSmad2/3) was found previously and evaluated in a phylogenetic analysis of the NvSmad family, but it has not been experimentally tested for function [2].

Experiments presented here test the abilities of *Nematostella* and *Drosophila* R-Smad orthologs to induce expression of downstream pathway genes and pattern tissues in the *Xenopus* embryo. We also probe the activities of individual Smad domains using chimeric constructs from *Xenopus* Smad2 and *Nematostella* Smad2/3. We find that cnidarian R-Smad proteins activate BMP and Activin/Nodal responses, but not at the efficiency of the native *Xenopus* proteins. However, we reveal qualitative differences in the ability of NvSmad2/3 to function in the developing vertebrate. Notably, vertebrate Smad2 and Smad3 have different signaling abilities, and only the bilaterian orthologs of Smad2/3 are capable of inducing ectopic axial structures in *Xenopus* embryos. Our findings show a deep conservation of fundamental Smad activities across 650 million years of animal evolution, but divergence in the smaller scale fine-tuning of gene activation, reflecting different evolutionary histories of the two major Smad TGFβ signaling pathways.

## Methods

### *Xenopus, Nematostella, and Drosophila* clones

The *Xenopus Smad1*, *Smad2*, and *Smad3* and *NvSmad1/5* clones were already available in the Thomsen Lab (Stony Brook University). *NvSmad2/3* was cloned directly out of cDNA prepared from total RNA of *Nematostella* planulae. The primers were designed from a predicted protein sequence [NCBI: XP_001631607], which was identified using a Basic Local Alignment Search Tool (BLAST) search with *XSmad2* sequence (forward primer: 5′ ATGACTTCCCTGTTGCCT 3′, reverse primer: 5′ CTACGATACCGAGGAGAT 3′). The PCR amplification was carried out with Platinum™ Taq DNA Polymerase High Fidelity ( Life Technologies, Invitrogen, Grand Island, NY). The PCR conditions were as follows: 94°C for 2 minutes (1 cycle); 94°C for 30 seconds, 56°C for 30 seconds, 68°C for 1.5 minutes (40 cycles); and 68°C for 2 minutes. The *Drosophila dSmad2* (or *Smox*) clone was a gift from the lab of Dr. Spyros Artavanis-Tsakonas (Harvard University) and the *Drosophila* Protein Interaction Map group. All clones were subcloned into the plasmid pCS2 containing three HA tags 5′ of the gene start site. The XSmad2ΔExon3 clone was a gift from the laboratory of Malcolm Whitman at Harvard University.

### Sequence analysis

Once subcloned, all clones were sequenced and checked against the correct protein sequence from GenBank. To create the alignments and pairwise comparisons used for Figure 1 and Additional file 1, we aligned the amino acid sequences by hand in MacVector (MacVector, Inc., Cary, NC), saved them as subdomain alignments, and opened them in ClustalW (European Bioinformatics Institute, Cambridge, UK, http://www.clustal.org) to calculate pairwise percent identity scores [see Additional file 2 for accession numbers].

### Chimera assembly

Amino acid boundaries for MAD Homology domains in XSmad2 and NvSmad2/3 are given in their entries at NCBI. MH1 chimera: [XSmad2: 1 to 172] + [NvSmad2/3: 131 to 423]. Linker chimera: [NvSmad2/3: 1 to 130] + [XSmad2: 173 to 267] + [NvSmad2/3: 224 to 423]. MH2 chimera: [NvSmad2/3: 1 to 223] + [XSmad2: 268 to 467]. In order to create the chimeric constructs, fragments were generated by PCR from *XSmad2* and *NvSmad2/3* clones [see Additional file 3 for primer locations and sequences]. The PCR amplification was carried out with Platinum™ Pfx DNA Polymerase from (Life Technologies). The PCR conditions were as follows: 94°C for 4 minutes (1 cycle), 94°C for 30 seconds, 55°C for 30 seconds, 68°C for 1 minute (35 cycles) and 68°C for 30 minutes. Primers were designed to amplify the desired region from one species and add approximately 10 nucleotides of the intended adjacent region of the other species, to generate fragments that would partially overlap within the chimeric product. Chimeric sequences were then generated by placing the appropriate fragments together in a PCR reaction and adding the primers corresponding to the ends of the desired chimeras. The fragments were ligated into pGEM-T vector and subcloned into an HA-tagged pCS2 vector. Chimeras were verified by sequencing.

### Messenger RNA synthesis

Clones were linearized and messenger RNA (mRNA) for microinjection was made from each clone using the Amplicap™ SP6 High Yield Message Maker kit (Epicentre Biotechnologies, Madison, WI). The mRNA was purified using a Qiagen RNeasy kit (Qiagen Inc., Valencia, CA), tailed using the Poly(A) Polymerase Tailing Kit (Epicentre Biotechnologies), and purified again before use.

### *Xenopus* embryo injections

*Smad1/5* phenotypes were generated by injecting 2 ng of mRNA (in 10 nl of nuclease-free water) into the marginal zone of both blastomeres at 4-cell stage (for a total of 4 ng). *Smad2/3* phenotypes were generated by injecting 0.5 ng (in 5 nl) into the marginal zone of one ventral vegetal blastomere at 8-cell stage [11,12,21]. Embryos were scored at neurula stage and allowed to grow until tadpole stage. Animal cap assays were performed by injecting 2 ng (in 10 nl) into the animal pole of each blastomere at 2-cell stage (for a total of 4 ng). All injections were performed in at least three different frogs and used for analysis. This research was compliant with the National Institutes of Health (NIH) Institutional Animal Care and Use Committee Guidelines and was approved by the Stony Brook University Internal Review Board.

### Translation assessment

Western blotting was performed to check for expression of the Heamaglutinin Antigen (HA) peptide tags and equalize translation levels. Embryos were lysed with a pipet tip in PBS 1% Triton at stage 11, at the same time as the animal caps from the same experiment were ready for harvesting. Lysates were spun at 4°, and soluble protein was mixed 1:1 with loading buffer and loaded in a 5% polyacrylamide gel. An Anti-HA primary antibody from Santa Cruz (sc-805) used at 1:500; the loading control was Abcam anti-β-Actin (ab 8229), used at 1:750. The secondary antibody was Alexa Fluor 680 goat anti-rabbit IgG from Life Technologies (A-21109), used at 1:10,000 [see Additional file 4 for full western blots and loading controls].

### *Xenopus* animal cap assay

Messenger RNA was injected into the animal pole of both blastomeres at 2-cell stage; animal caps were harvested at stage 8 and cultured in 0.5× Marc’s Modified Ringers (MMR) buffer until stage 11. Cells were lysed with Proteinase K and total RNA was extracted from the animal caps and whole embryo controls using phenol:chloroform extraction, followed by ethanol precipitation. Next, cDNA was synthesized using 1 μg of total RNA and SuperScript II Reverse Transcriptase enzyme from Invitrogen (Life Technologies). Then, cDNA samples were analyzed on a Roche Diagnostics LightCycler 480 System using SYBR™ Green Mastermix I from Roche Diagnostics (Indianapolis, IN). Animal cap cDNA was compared to cDNA from a whole embryo, representing the endogenous expression levels. For each primer pair in each experiment, serial dilutions of whole embryo cDNA were used to create the standard curve to which all samples were compared in order to calculate concentration of PCR product. Once concentrations were acquired and imported into Excel, raw values were normalized to the level of Ornithine Decarboxylase (ODC), a housekeeping gene. See Additional file 5 for a table of LightCycler primer sequences and quantitative RT-PCR (qPCR) conditions, and their references.

## Results and discussion

### *Nematostella* Smads contain the highly conserved MAD-homology domains that define bilaterian Smads

First, we revisited the presence and identities of R-Smads in *Nematostella*. Previous work identified one AR-Smad (NvSmad2/3) and one BR-Smad (NvSmad1/5) [2,3], and our re-examination of genomic and cDNA sequences confirmed those earlier identifications, but since the NvSmad2/3 ortholog was only reported as a predicted protein [NCBI:XP_001631657], we isolated a full-length copy of this cDNA (see Methods). We then performed pairwise alignments of all R-Smad orthologs from *Xenopus* and *Nematostella* to validate their relationships and highlight their unique features [see Additional file 1 and Additional file 2 for detailed alignments and accession numbers].

We found that the amino acid sequences of the MAD homology domains are highly conserved between *Xenopus* and *Nematostella* (Figure 1B). The N-terminal MH1 DNA-binding domain is more conserved in the Smad1/5 category (86%) than in the Smad2/3 category (78 to 79%). The C-terminal MH2 protein-interacting domain is the most conserved in each R-Smad category, and is equally conserved between Smad1/5 and Smad2/3 (88 to 89%). The linker region is less conserved than the MAD homology domains, 20% in Smad1/5 and 33 to 34% in Smad2/3. Since the linker region is more variable yet contains important sites for post-translational regulation, we performed a second, more inclusive alignment of linker domains in order to investigate the status of several important sites. We included R-Smad orthologs from the human and from *Drosophila melanogaster* in this part of this analysis [see Additional file 2 for accession numbers]. Figure 1C and D show alignments of the important residues of the linker regions.

The human Smad1/5/9 linker contains four conserved proline-X-serine-proline (PXSP) consensus sites for MAPK phosphorylation [22], which are putatively present in *Xenopus* Smad8a and 8b (Figure 1C, yellow). The *Drosophila* dMad linker contains two conserved MAPK sites (Figure 1C, underline and yellow) [23], and the NvSmad1/5 linker shows one potential site (Figure 1C). With the exception of human Smad9b, vertebrate and *Drosophila* Smad1/5/8 orthologs share the PPXY motif that binds Smurf1, an E3 ubiquitin ligase that, once bound, will bring about ubiquitin-mediated degradation of these Smads [24] (Figure 1C, cyan). The linker of NvSmad1/5, however, lacks this site (Figure 1C). The dMAD linker also contains eight serine/threonine phosphorylation sites for GSK3 [23], which show variable conservation in the other orthologs (Figure 1C, green). The vertebrate orthologs contain seven of these predicted sites, and the linker of NvSmad1/5 contains potentially five of them.

The human Smad2 and Smad3 orthologs contain a MAPK consensus site [25] that is also found in *Xenopus* orthologs, putatively in dSmad2, and partially in NvSmad2/3 (Figure 1D, yellow). With the exception of NvSmad2/3, the linkers of all Smad2/3 orthologs possess a PPXY motif, which allows targeting by Smurf2 for ubiquitin-mediated degradation [26] (Figure 1D, cyan). The human Smad2 and Smad3 orthologs contain three serine/proline phosphorylation target residues [27,28] that are present in the *Xenopus* and *Drosophila* orthologs, and two of which appear in NvSmad2/3 (Figure 1D, magenta). These analyses illustrate that cnidarian R-Smad linker regions may have fewer points of regulation compared to bilaterian R-Smads, suggesting that NvSmad1/5 could be regulated in a different manner from bilaterian orthologs.

### Overexpression of *NvSmad1/5* causes ventralization phenotypes in *Xenopus* embryos

**Figure 2:**
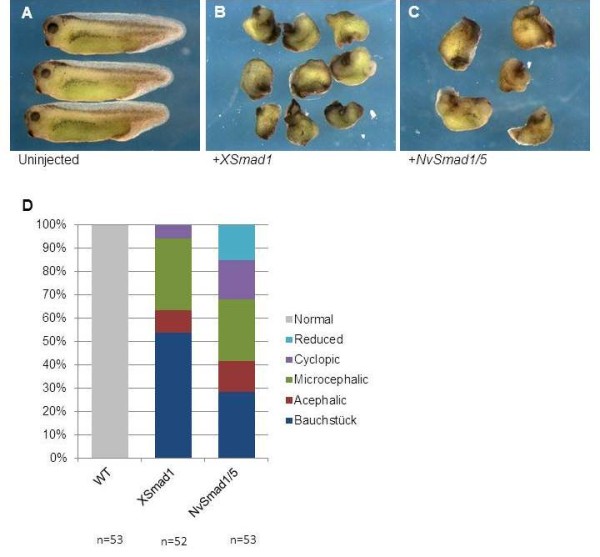
**Phenotypes caused by ectopic *NvSmad1/5 *mRNA mimic phenotypes caused by *XSmad1 *mRNA.** Microinjection of *NvSmad1/5* mRNA into the two dorsal blastomeres of a four-cell *Xenopus* embryo causes similar ventralization phenotypes as injection of *XSmad1* mRNA. All tadpoles are shown at stage 34. (**A**) Shows normal development in *Xenopus* tadpoles. In contrast, tadpoles in (**B**) were injected with 4 ng of *XSmad1* mRNA and show severe ventralization. (**C**) Tadpoles injected with 4 ng *NvSmad1/5* show a similar but less severe ventralization phenotype. Severity of phenotype was scored according the Dorso-Anterior Index (DAI) [30]. (**D**) Shows the DAI scores graphically.

Bilaterian BR-Smad orthologs can ventralize *Xenopus* embryos when ectopically expressed in dorsal tissues. We tested whether NvSmad1/5 could function similarly when ectopically expressed *in vivo* in *Xenopus* embryos. We compared the phenotype from ectopic expression of NvSmad1/5 to that of XSmad1 (the *Smad5* gene is not present in *Xenopus laevis*, and *XSmad8* transcripts are scarce during gastrulation [29]). We found that ectopic dorsal expression of NvSmad1/5 generated the hallmarks of BMP overexpression: ventralization and obliteration of head structures. By stage 34, uninjected wild type tadpoles had obvious head and neural structures (Figure 2A), whereas tadpoles that had been injected with *XSmad1* mRNA showed a range of ventralization phenotypes, the most severe of which are shown in Figure 2B. Injection of *NvSmad1/5* mRNA also showed a range of ventralization effects, the most severe of which are shown in Figure 2C.

To quantify the range of effects, we used Kao and Elison’s DorsoAnterior Index (DAI) to score the severity of the ventralization phenotypes on a scale of 0 (most severely ventralized) to 5 (normal) [30]. Overall, the XSmad1 phenotypes scored as more severe than the NvSmad1/5 phenotypes (Figure 2D). The weighted means of the XSmad1 and NvSmad1/5 phenotypes were 0.89 and 1.77, respectively. The standard deviation of the XSmad1 scores was less than that of the NvSmad1/5 scores, 1.0 and 1.4 respectively. The XSmad1 overexpression phenotype is overall more severe and has less range, whereas the NvSmad1/5 phenotype is less severe and shows more variation. These results indicate that the NvSmad1/5 protein functions in the *Xenopus* embryo and successfully generates the expected ventralization effects of BMP activity, but it is less potent than the native XSmad1 protein under the same conditions.

### NvSmad1/5 induces downstream *BMP* marker gene expression in X*enopus*

**Figure 3:**
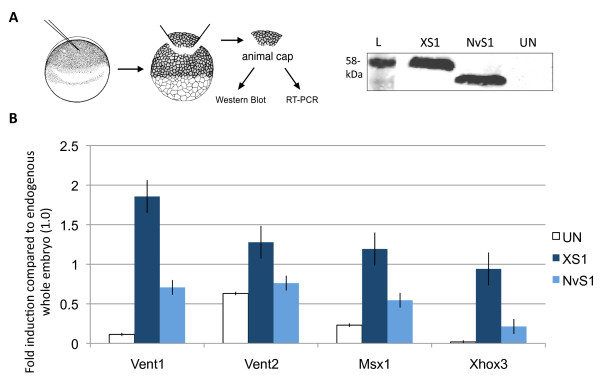
***NvSmad1/5 *induces expression of downstream *BMP *pathway genes in the frog embryo.** After animal pole injection of *XSmad1* or *NvSmad1/5* at the 2-cell stage, stage 11 animal caps show elevated expression of genes downstream of the BMP pathway. (**A**) Depiction of the animal cap assay procedure; animal caps were processed for western blot or RT-PCR when control embryos reached mid-gastrulation (Niewkoop and Faber stage 11). The western blot shows protein translation levels in injected and uninjected whole embryos (L = Benchmark protein ladder, showing the 58-kDa band). XSmad1 and NvSmad1/5 show equal levels of translation, whereas the uninjected embryos show no background signal. (**B**) Real-time quantitative RT-PCR (qPCR) shows fold induction levels of BMP pathway response genes *Vent1, Vent2, Msx1,* and *Xhox3* compared to the uninjected whole embryos. Uninjected *Xenopus* animal caps (UN), animal caps injected with *XSmad1,* and animal caps injected with *NvSmad1/5* are shown. The Y-axis of all RT-PCR graphs shows the fold induction compared to endogenous whole embryo (1.0). Error bars indicate the standard error.

The observation that ectopic expression of NvSmad1/5 and XSmad1 results in similar ventralization phenotypes led us to compare their inductive activity more precisely, and determine whether NvSmad1/5 has the ability to initiate similar downstream gene expression in *Xenopus*. To do this, we used *Xenopus* animal cap assays to compare the expression levels of ventral marker genes known to be downstream of BMP signaling. We used tagged expression vectors and western blotting to confirm equal protein translation levels before performing RT-PCR analysis (Figure 3A) [see Additional file 4 for western blot loading controls].

In three out of four cases, NvSmad1/5 induced expression at a level significantly higher than that of the uninjected animal caps (Figure 3B). NvSmad1/5 was able to induce downstream BMP pathway members *Vent1, Msx1,* and *Xhox3* at levels higher than in uninjected animal caps, yet at roughly half the levels induced by the native XSmad1 protein. However, in all cases, NvSmad1/5 failed to induce expression equal to endogenous levels in the whole embryo (set as 1.0 on the Y-axis for all RT-PCR analyses). We were not able to see a clear induction response by *Vent2*, which may be due to high levels of endogenous *Vent2* expression. Thus, despite the absolute differences in activity between NvSmad1/5 and XSmad1, NvSmad1/5 can initiate transcription of *Xenopus* BMP target genes.

### *NvSmad2/3* induces expression of a subset of markers of the Activin/Nodal pathway

**Figure 4:**
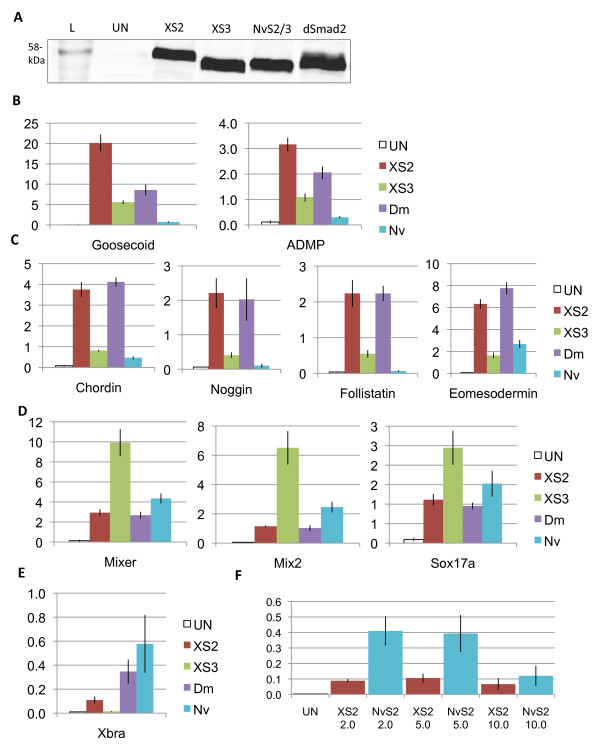
***NvSmad2 *induces expression of downstream Activin/Nodal pathway genes in *Xenopus *.** Animal caps injected with *XSmad2, XSmad3, dSmad2,* or *NvSmad2* mRNA at the two-cell stage show elevated expression of genes downstream of the Activin/Nodal pathway. (**A**) Western blot showing tagged protein translation levels in injected and uninjected whole embryos (L = Benchmark protein ladder, showing the 58-kDa band). XSmad2, XSmad3, NvSmad2/3, and dSmad2 show equal levels of translation, whereas uninjected embryos show no background signal. Real-time quantitative PCR (RT-PCR) shows fold induction levels of Activin/Nodal pathway members on the Y-axis. (**B**) Class I markers *goosecoid* and *anti-dorsalizing morphogenetic protein* (*ADMP*). (**C**) Class II markers *chordin*, *noggin*, *follistatin*, and *eomesodermin*. (**D**) Class III markers *mixer*, *mix.2*, and *sox17α*. (**E**) Class IV marker *Xbra*. (**F**) *Xbra* induction response to 2 ng, 5 ng, and 10 ng *XSmad2* or *NvSmad2/3.* See text for discussion of marker classes.

In order to test the functional conservation of vertebrate and cnidarian AR-Smad orthologs, we examined the ability of NvSmad2/3 to initiate Activin/Nodal signaling in the *Xenopus* animal cap. Equal protein translation levels were confirmed using western blotting before RT-PCR analysis (Figure 4A) [see Additional file 4 for western blot loading controls]. Unlike the uniformity of marker induction by NvSmad1/5, the induction response to XSmad2 and NvSmad2/3 showed two clear patterns: for some markers NvSmad2/3 showed only a fraction of the inductive power of the native XSmad2, whereas for other markers, NvSmad2/3 was equal to or greater than XSmad2 in its inductive abilities (see Figure 4B-E red and teal bars).

To investigate these patterns, we included additional AR-Smad orthologs. We chose the *Drosophila* AR-Smad dSmad2 as a protostome representative and XSmad3 as the second vertebrate AR-Smad ortholog. Upon repeating these experiments with all four treatments, further trends became evident. We were able to split Activin/Nodal markers into four classes based upon their inductive response. Class I included *goosecoid* and *ADMP*, two genes expressed strictly in the Spemann organizer of the developing amphibian. Both of these were strongly induced by XSmad2 and less so by the other orthologs (Figure 4B). Class II markers were induced strongly by XSmad2 and dSmad2, and responded poorly to XSmad3 and NvSmad2/3 (Figure 4C). Class II included three BMP-inhibitors - *chordin, noggin,* and *follistatin*, as well as *eomesodermin*, another gene associated with dorsalization. In contrast, Class III markers were induced strongly by XSmad3, while XSmad2, NvSmad2/3, and dSmad2 showed relatively less response (Figure 4D). Class III markers are more general mesendoderm-related Activin/Nodal markers *mix2, mixer,* and *sox17α*.

*Xbrachyury* was in a class by itself, Class IV (Figure 4E). *Xbra* induction by Smad2/3 orthologs was generally low. The highest induction was by NvSmad2/3 and reached almost 60% of endogenous level in the *Xenopus* embryo (1.0 on the Y-axis in all RT-PCR analyses). To test whether we were experimenting at the appropriate dosage (4 ng), we compared three different dosages of NvSmad2/3 and XSmad2 - 2 ng, 5 ng, and 10 ng. Results were similar; NvSmad2/3 induced more strongly, while XSmad2 induced very weakly (Figure 4F). *Xbra* response to the lower doses of NvSmad2/3 remained consistent with previous results, while *Xbra* response to the highest dose of NvSmad2/3 dropped to the low level of *Xbra* response to XSmad2.

### Substituting the NvSmad2/3 MH2 with the XSmad2 MH2 increases inductive capability

**Figure 5:**
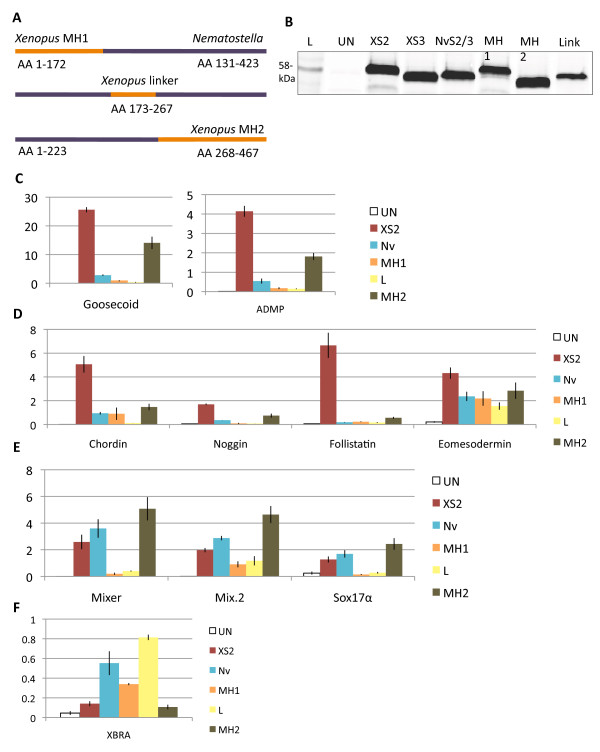
**Swapping in the MH2 domain from *XSmad2 *increases the inductive activity of *NvSmad2/3 *.** (**A**) Design of chimeras swapping *XSmad2* domains into *NvSmad2/3*. (**B**) Western blot showing tagged protein translation levels in injected and uninjected whole embryos (L = Benchmark protein ladder, showing the 58-kDa band). XSmad2, XSmad3, NvSmad2/3, MH1 chimera, and MH2 chimera show equal levels of translation, whereas the uninjected embryos show none. The linker chimera (‘Link’) shows slightly lower levels of translation. Note, *XSmad3* was injected and included in translation blot to check continuity with previous experiments, but was not used in subsequent animal cap analyses. Animal caps injected with *XSmad2, NvSmad2,* MH1 chimera, linker chimera, or MH2 chimera mRNA at the two-cell stage show elevated expression of genes downstream in the Activin/Nodal pathway. Real time quantitative PCR shows fold induction levels of Activin/Nodal pathway members on the Y-axis. (**C**) Class I markers *goosecoid* and *ADMP*. (**D**) Class II markers *chordin*, *noggin*, *follistatin*, and *eomesodermin*. (**E**) Class III markers *mixer*, *mix.2*, and *sox17α*. (**F**) Class IV marker *Xbra*.

The Smad2/3 orthologs showed very particular induction patterns in our *Xenopus* animal cap assays. We wished to determine whether the differences in activity between XSmad2 and NvSmad2/3 might reflect evolutionary specialization of specific regions of XSmad2, particularly whether any single domain from XSmad2 could increase the capability of NvSmad2/3 to induce organizer markers in *Xenopus.* To this end, we created three chimeras that replaced the domains in NvSmad2/3 one at a time with XSmad2 domains (Figure 5A), and tested their inductive abilities in animal cap assays with the same set of markers as above. We confirmed equal translation levels with western blotting before RT-PCR (Figure 5B) [see Additional file 4 for western blot loading controls]. The linker chimera (‘Link’ in Figure 5B) showed a slightly lower amount of protein than the others at 4 ng mRNA injection. It remained at a lower level even at 8x the injection concentration of the other treatments (data not shown), so we kept the injection concentrations equal.

Interestingly, the four classes of markers from our previous experiment were largely consistent in this experiment as well. In Class I markers *goosecoid* and *ADMP*, substitution of the XSmad2 MH2 domain (“MH2 chimera”) led to a gain in inductive ability over the wild type NvSmad2/3, to about 50% of the level of XSmad2 induction (Figure 5C). For Class II markers *chordin*, *follistatin*, and *eomesodermin*, the MH2 chimera showed very slight enhancement in inductive ability, but that was still only a fraction of the level of induction observed with XSmad2 (Figure 5D). For Class III markers, NvSmad2/3 inductive ability was already slightly higher than that of XSmad2, and the MH2 chimera showed a modest increase (Figure 5E). For *Xbra*, the Class IV marker, the MH2 chimera had significantly less inductive activity than NvSmad2/3 (Figure 5F).

In all cases, substitution of the XSmad2 MH1 domain (‘MH1 chimera’) had a negative effect on the inductive capacity of NvSmad2/3 (Figure 5C-F). Likewise, swapping in the XSmad2 linker region for the NvSmad2/3 linker region (‘linker chimera’) resulted in a drop in inductive ability of nearly every marker tested. Again, *Xbra* showed its own unique response pattern; it was the only marker to respond more strongly to the linker chimera than to the wild type NvSmad2/3 (Figure 5F). The *Xbra* response levels to wild type XSmad2 and NvSmad2/3 correspond to our previous dosage observations (Figure 4E).

### NvSmad2/3 does not induce the formation of a second body axis when ectopically expressed in *Xenopus* embryos

NvSmad2/3 shows a complicated activity pattern in regard to its induction of dorsal mesoderm markers and Activin/Nodal targets. This calls into question the level of Smad2/3 functional conservation within Metazoa. It has been shown previously that Smad2 from the mouse can induce a second body axis in *Xenopus* embryos [31], one with trunk and tail characteristics but lacking a head. This is nearly identical to axial structures induced by ectopically-expressed *Xenopus* activin [32] and indicates that Smad2 function is conserved among vertebrates. We performed ectopic expression experiments to determine whether the ability to induce a second body axis is unique to the vertebrate Smad2 ortholog. Alternatively, that ability could be inherent to both of these vertebrate Smad2/3 paralogs, to all bilaterian Smad2/3 orthologs, or more generally to all metazoan Smad2/3 orthologs.

**Figure 6:**
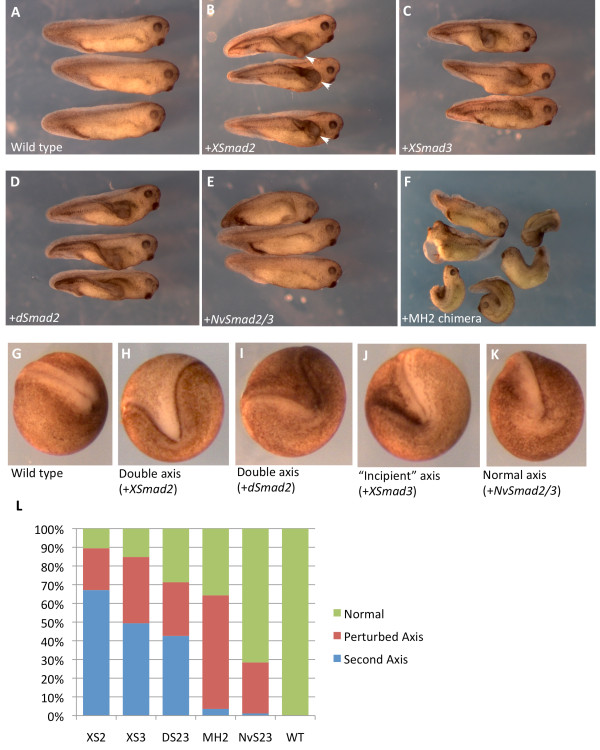
**Ectopic overexpression of *NvSmad2/3 *does not induce a secondary axis in *Xenopus *embryos.** Injection of 0.5 ng *XSmad2* mRNA into the marginal zone of one ventral vegetal blastomere at the 8-cell stage can produce a secondary body axis in *Xenopus* embryos. (**A**) Uninjected, wild type tadpoles. (**B**) Tadpoles that were injected with *XSmad2* show a classic secondary body axis phenotype (marked with white arrowheads in this photo only). (**C**) Injection of *XSmad3* shows a clear secondary axis. (**D**) *dSmad2* is able to induce the formation of a second body axis. (**E**) *NvSmad2/3* is not able to generate a second body axis, but can perturb the original axis. (**F**) The MH1 chimera acutely perturbs the original axis, but generates a complete second axis in only a few cases. Embryos were scored for axial phenotypes at neurula stage. Examples: (**G**) wild type, (**H**) double axis that would result in a second body axis at tadpole stage (result of *XSmad2* mRNA, in this case), (**I**) another double axis (caused by *dSmad2* mRNA, in this case), (**J**) ‘incipient’ axis that will eventually get subsumed into the primary axis and result in the ‘perturbed axis’ phenotype (result of *XSmad3* mRNA, in this case, though it could be caused by any of the treatments), (**K**) phenotype that would be scored as ‘wild type’ (result of *NvSmad2/3* mRNA, in this case). (**L**) Bar graph illustrating the range of phenotypes from each treatment. See Additional file 6 for more photos illustrating the ‘perturbed axis’ phenotype.

We observed a very strong secondary axis phenotype caused by bilaterian Smad2/3 orthologs (Figure 6A-D). The secondary axis was evident as a second set of neural folds at neurula stage (Figure 6G-K) and developed into an unmistakable secondary trunk by tadpole stage (Figure 6B, white arrowheads). XSmad2 produced a secondary axis in 65% of embryos, whereas XSmad3 did so in about 50% of embryos, and dSmad2 in 45% (Figure 6L). In another 25 to 35% of cases, both proteins did not generate a distinct secondary axis, but did create a small “incipient” second axis at the neurula stage (for example, Figure 6J) that was subsumed into the primary axis during development and eventually manifested as the ‘perturbed’ axis of the tadpole [see Additional file 6].

NvSmad2/3 did not effectively produce a secondary axis, but it did perturb the primary axis in 25% of embryos (Figure 6E). NvSmad2/3 did appear to generate a secondary body axis in one embryo (n = 88), but it was from a relatively unhealthy batch of embryos and this example was not representative of the overall performance of NvSmad2/3. The MH2 chimera did not improve upon the ability of NvSmad2/3 to produce a secondary body axis, but it perturbed the natural axis in upwards of 50% of embryos (Figure 6F, L).

These data agree with other data we present here that suggest that bilaterian Smad2/3 orthologs have developed functions that non-bilaterian orthologs are unable to perform *in vivo*. These data also support our results indicating that swapping XSmad2 domains onto NvSmad2/3 cannot bestow full functional abilities.

### NvSmad1/5, but not NvSmad2/3, can recapitulate activity of bilaterian orthologs

NvSmad1/5 engaged the *Xenopus* pathway well enough to cause very severe ventralized phenotypes (Figure 2) and activate transcriptional targets (Figure 3), although at a lower level than XSmad1. We found that ectopic expression of NvSmad2/3 was unable to induce a secondary axis in *Xenopus* embryos, and showed differences in downstream induction of Activin/Nodal markers when compared to XSmad2, including the BMP inhibitors *noggin*, *chordin*, and *follistatin*, and the organizer-specific genes *goosecoid* and *ADMP.* All of these except *ADMP* are known to have cnidarian orthologs [33]. Interestingly, NvSmad2/3 induced the general mesendoderm markers at the same level as some of the bilaterian orthologs (Class III, Figure 4D). There is no ortholog of *nodal* known in *Nematostella*, but *NvActivin* is expressed in the endoderm during gastrulation [33]. Likewise, the *Sox17* ortholog *NvSoxF1* is expressed broadly in the endoderm following gastrulation (there are no definitive orthologs of *mix2* or *mixer* yet known to be expressed in developing *Nematostella* endoderm) [34]. Our data are further evidence that *Activin* signaling via AR-Smads to pattern endoderm is an ancient and conserved mechanism in metazoan development.

One alternative explanation for the differential activation of gene targets by NvSmad2/3 in our experiments could be a dose-dependence. Experiments incubating *Xenopus* animal caps with Activin ligand have revealed striking dose-dependent induction of mesodermal markers including *Xbra* and *goosecoid* by Activin, which are activated at low and high doses of Activin respectively [35,36]. We observed a concordant *Xbra* dose-dependent response to ligand-independent overexpression of either *Xenopus* or *Nematostella* Smad2/3 (Figure 4F and results not shown). We reasoned that if the particular dose of Smad2/3 was responsible for these differences in gene induction, then programming the animal cap system with graded concentrations of NvSmad2/3 (up to 10 ng) might yield sufficient activity to replicate the inductive patterns observed with XSmad2 (for example, induction of Xbra and Xgsc at respectively low and high levels of NvSmad2/3). To the contrary, however, the response patterns of most markers remained consistent for all three doses tested (Additional file 7). Increasing the level of NvSmad2/3 to 10 ng did not activate the *goosecoid* gene even to a level induced by the lowest amount of XSmad2 (see Additional file 7).

We propose that the differences in cnidarian versus bilaterian Smad2/3 activity reflect evolutionary divergence, which has rendered NvSmad2/3 unable to engage the necessary signaling, transcriptional, or other necessary cofactors in the *Xenopus* system. This may be due to lack of key microdomains or amino acid residues that are present in *Xenopus* and other bilaterian Smad2/3 orthologs which facilitate more efficient or complete engagement and activation of target genes. For instance, Smad2 and Smad3 proteins make complexes with Smad4, FAST-1, p53 and other co-factors in order to enter the nucleus, bind DNA, and transcribe target genes [13,35,37]. The low inductive activity of NvSmad2/3 in *Xenopus* could be due to NvSmad2/3 forming transcriptional complexes that are weak, unstable, and/or inactive. Smads are also a common target of TGFβ signal regulation by other pathways, such as FGF (via MAPK) and Wnt (via GSK3) [36], thus there are various ways in which the subtle protein sequence differences between NvSmad2/3 and vertebrate Smad2 and 3, especially those in the linker domain, could lead to differences in activity.

Despite the low inductive ability of NvSmad1/5 relative to XSmad1, it could still re-pattern the *Xenopus* embryo to cause severe significant ventralization of dorsal tissues. This was not the case with NvSmad2/3, which could not induce the secondary body axis observed with overexpression of XSmad2*,* XSmad3*,* or dSmad2 (Figure 6E, G). Mouse Smad2 can also generate a very pronounced second axis in *Xenopus* embryos [31], which builds the case that bilaterian Smad2/3 orthologs have a function that the non-bilaterian NvSmad2/3 is not able to perform. This suggests fine-scale divergence in the case of Smad1/5 and larger-scale divergence in the evolutionary history of Smad2/3*.*

### Vertebrate Smad2 and Smad3 have different activity

There are numerous indicators that vertebrate Smad2 and Smad3 have different activities. There is evidence of exclusive co-factors for each in zebrafish [38], and vertebrate Smad2 and Smad3 differ in their mechanisms of nuclear import and their regulation by ubiquitination [8,13,26,39]. Their divergent gene induction activities in our animal cap assays also suggest a division of labor. Most significantly, XSmad2 shows greater transactivation of markers associated with the Spemann organizer, particularly genes encoding dorsalizers such as the BMP inhibitors *chordin*, *noggin*, and *follistatin*. XSmad3, on the other hand, is more efficient in the activation of general mesendodermal genes such as *mix2 and mixer,* and the endoderm-specific gene *sox17α* (Figure 4C). This division of labor agrees with the observations that Smad3 might be more involved in TGFβ-mediated cell cycle control in some cell lines, reflected by the findings that mutations in Smad3 are more prevalent in some types of cancer [13]. Mouse gene knockout phenotypes also indicate that Smad2 may have a greater role than Smad3 during embryonic development, with Smad3 contributing more to the regulation of cell stasis [15].

NvSmad2/3 has comparable inductive ability to XSmad3 (stronger with mesendodermal genes, weaker with organizer-related genes), whereas XSmad2 and dSmad2 show similar inductive ability (stronger with organizer-related genes, weaker with mesendodermal genes). This makes it tempting to propose that XSmad3 retains deep ancestral function similar to NvSmad2/3; however, functional testing showed that XSmad3 produces a secondary body axis in the same manner as XSmad2 and dSmad2, while NvSmad2/3 does not (Figure 6L). This creates a very complicated picture of Smad3; it has the ability to control the embryonic organizing center and induce dorsal tissue fates as well as Smad2, but *in vitro* it shows more affinities for induction of mesendoderm-related genes. We infer that the Smad2/3 progenitor may have acquired its ability to control the evolving vertebrate organizer before the duplication event, and that the ‘division of labor’ after the duplication event appears to be superficial, affecting the protein’s activity rather than its actual function.

**Figure 7:**
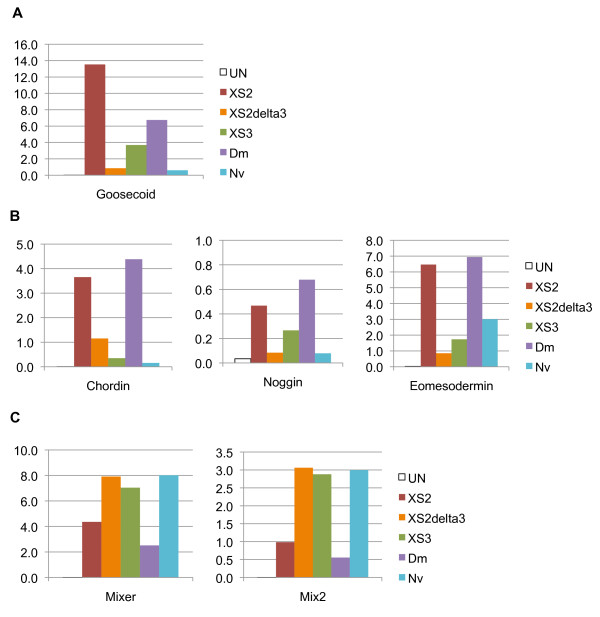
**XSmad2ΔExon3 induces expression of downstream Activin/Nodal pathway genes in a pattern resembling XSmad3 rather than full-length XSmad2.** Animal caps from gastrulae that had been injected with *XSmad2, XSmad2ΔExon3, XSmad3, dSmad2,* or *NvSmad2* mRNA at the two-cell stage showed elevated expression of genes downstream of the Activin/Nodal pathway. With all markers, the expression patterns induced by XSmad2ΔExon3 (orange column) were more similar to that of XSmad3 (green column) and NvSmad2/3 (teal column) than to full length XSmad2 (red column). (**A**) Class I marker *goosecoid*. (**B**) Class II markers *chordin*, *noggin*, and *eomesodermin*. (**C**) Class III markers *mixer* and *mix.2*.

One important contributor to this division of labor between vertebrate Smad2 and Smad3 may have been the evolution of exon 3 in vertebrate Smad2. This exon encodes a 30-amino acid insertion positioned within the MH1 domain immediately adjacent to the predicted DNA-binding hairpin [see Additional file 1. This insertion prevents proper DNA binding by Smad2, but Smad3, lacking this insert, binds DNA. Interestingly, an alternatively spliced version of Smad2 mRNA encodes a protein that does not include exon 3 (known as Smad2ΔExon3) and this variant of Smad2 has been shown to bind to DNA [40]. Smad2ΔExon3 splice variant transcripts and protein have been found in gastrula stage *Xenopus* embryos [41], and various mammalian cell lines. We have tested the ability of *Xenopus* Smad2ΔExon3 to activate Activin/Nodal signaling markers, and our results indicate that the activity of XSmad2ΔExon3 is, more similar to that of XSmad3 and NvSmad2/3 than it is to XSmad2 (Figure 7). The functional importance of exon 3 in Smad2 signaling, and its origin during vertebrate evolution merits further analysis in the future.

### The MH2 domain has the largest influence on R-Smad induction capability

The results of our chimeric R-Smad analysis underscore the importance of the MH2 domain as a determinant of gene activation, and illustrate an interesting aspect of sequence conservation versus signaling activity. The MH2 domain is the most conserved protein domain between R-Smad orthologs from various species (Figure 1B) [see Additional file 1, yet despite this high degree of sequence conservation, replacement of the MH2 domain in NvSmad2/3 with the XSmad2 MH2 shows the greatest enhancement of NvSmad2/3 activity (Figure 5C, D). This points to the importance of the few amino acid residues that vary between the MH2 domains of *Xenopus* and *Nematostella* proteins, which may not be revealed by natural mutagenesis (for example, cancer mutations) or directed changes. These types of substitutions are most frequently reported in the MH2 when they have a significant effect on Smad signaling, such as those of the loop-strand pocket that are involved in receptor docking and specificity [42], those in the co-factor binding hydrophobic pocket [43,44], or those essential to Smad trimerization [45,46]. Our observed patterns of differential downstream gene induction between species are more subtle than these large effects, and indeed, in the great majority of cases, residues that are reported to be functionally important are conserved across species (Additional file 1). To reveal which residues contribute to the induction patterns reported here, we suggest further experimentation with chimeric constructs, especially single amino acid replacements of positions known for greater variability.

In contrast to MH2, the MH1 chimera did not improve the signaling capacity of wild type NvSmad2/3 (Figure 5C-E). One likely reason for this is that the vertebrate Smad2 MH1 domain lacks the ability to bind DNA. As noted above, vertebrate Smad2 differs from Smad3 and all other Smad2/3 orthologs due to the 30-amino acid insert (coded by exon 3) preceding the DNA binding domain of the MH1 between the L2 loop and the β-hairpin (see Additional file 1). In Smad4, mutating amino acids in this region severely disrupts DNA binding [40,47], and deletion of exon 3 from XSmad2, in the natural splice variant XSmad2ΔExon3 significantly altered its signaling activity in animal caps (Figure 7). Besides the exon 3 insert in XSmad2, the first five amino acids of the L2 loop itself are different in NvSmad2/3 and XSmad2*.* It would be informative to swap the XSmad3 or NvSmad2/3 MH1 domains separately onto XSmad2 in order to restore DNA binding ability and test whether there is a difference in downstream gene expression or ability to induce a second axis by XSmad2.

In general, replacing the NvSmad2/3 linker region with that of XSmad2 decreased its inductive ability. Given the low protein level of the linker chimera relative to the other Smad2/3 proteins we assayed (Figure 5B, last column), the XSmad2 linker domain may destabilize the NvSmad2/3 protein structurally or by introduction of additional sequences that direct post-translational modifications. The NvSmad2/3 linker lacks motifs that are essential for these regulatory processes (Figure 1D), including a proline-proline-X-tyrosine (PPXY) consensus motif targeted by Smad ubiquitin-ligases such as Smurf2 [26,48]. Interestingly, we were unable to identify clear Smurf1 or Smurf2 orthologs in the *Nematostella* genome or ESTs, which appears to correspond to the absence PPXY motifs in either *Nematostella* Smad. Addition of the *Xenopus* linker is predicted to cause NvSmad2/3 to undergo a more complex level of regulation *in vivo* in *Xenopus* embryos than wild type NvSmad2/3 might in the sea anemone, likely making the chimera sensitive to Smurf2 or NEDD4-L mediated ubiquitylation and degradation.

Despite its apparent lack of activity on many endogenous *Xenopus* genes, the linker chimera induced downstream Activin/Nodal target genes *eomesodermin*, *mix.2*, and *Xbra* at levels that approach or exceed those observed in the uninjected whole embryo (Figure 5D, E). This indicates that the linker chimera is not simply nonfunctional, but instead that its unique combination of sequence features renders it suited to induce only a subset of Activin/Nodal response genes. To address this possibility, it would be interesting to point-mutate some of the specific kinase target residues in the NvSmad2/3 linker to create sites that confer vertebrate-like linker regulation, and test the activities of such mutants. This would help distinguish the effects of linker-driven posttranslational regulation from transcriptional activity of the *Nematostella* nd *Xenopus* proteins. Conversely, it would be interesting to replace the XSmad2 linker with that of NvSmad2/3 and test whether the decrease in linker regulation sites has any effect on the ability of XSmad2 to activate target marker genes. Our results raise interesting questions about the evolution of R-Smad functions during metazoan diversification. For example, we would like to understand how differences in R-Smad protein sequences correlate with the acquisition or loss of target genes (and protein cofactors) among testable species in major taxonomic clades, particularly at nodes where Smad gene duplications have occurred or where Smad signaling pathway complexities have been streamlined by genome reduction. This would require a greater breadth of *in vivo* functional tests, assaying activities of orthologous Smads between species. A desirable next extension of the present study would be to test wild-type orthologs and chimeric R-Smads in *Nematostella* embryonic assays (and ideally *Drosophila* embryos as well). Such tests would provide additional information about the evolution of Smad structure and function as well as provide important information about the biological actions of Smad signals in cnidarian germ layer specification and cell fate determination.

## Conclusions

In this study we compared and contrasted the signaling activities of the two R-Smads of *Nematostella* with their bilaterian orthologs, in the context of a developing vertebrate. We find that the BMP-specific R-Smad, NvSmad1/5, can pattern (ventralize) the mesoderm of *Xenopus laevis* embryos and activate downstream genes in a similar, albeit less efficient, manner than a vertebrate ortholog, *Xenopus* Smad1. This speaks to a deep conservation of function within the BMP pathway of bilaterians and earlier-diverging metazoan groups. Further, we find that the Activin R-Smad, NvSmad2/3, is a strong inducer of mesendodermal and definitive endoderm genes, suggesting that the development of endoderm via Smad2/3 signaling is also an ancient and conserved system. However, the cnidarian NvSmad2/3 fails to induce a secondary body axis in *Xenopus* embryos and is inconsistent in its ability to activate downstream target genes compared to its bilaterian counterparts XSmad2, XSmad3, and the sole *Drosophila* AR-Smad, dSmad2.

Based on our results and previous reports, we propose that the bilaterian ancestor solidified a novel role for the Smad2/3 ortholog in controlling body patterning that the NvSmad2/3 is unable to perform. Furthermore, our animal cap assays are the first to test the inductive activities of Smad2 and Smad3 side by side, and indicate different target gene affinities for the two, with XSmad2 having substantially greater effects on organizer-specific genes than general mesendodermal genes, whereas XSmad3 displays converse actions. This demonstrates an intriguing division of labor that leads us to suggest that vertebrate Smad2 has evolved novel activities that govern the vertebrate organizer. Compellingly, the division of labor between these duplicates is relatively “superficial,” being that both vertebrate AR-Smads and the *Drosophila* ortholog dSmad2 are capable of patterning dorsal tissues and inducing a secondary axis in *Xenopus* embryos.

The MH2 domain has a major influence on AR-Smad inductive capability, yet this domain is 96% identical in XSmad2 and XSmad3, highlighting the importance of particular residues whose random mutation is not lethal to the organism, but may instead bring about slight functional changes that can be selected on and affect evolutionary divergence. Activity tests on a more comprehensive set of R-Smad orthologs gathered from major taxonomic groups should be very informative about the evolution of R-Smad structure/function and target gene regulation.

## Abbreviations

ADMP: Anti-dorsalizing morphogenetic protein; AR-Smad: Activin-Nodal Pathway smad; BMP: Bone morphogenetic protein; BR-Smad: an R-Smad in the BMP pathway; Co-Smad: Common smad; DAI: DorsoAnterior Index; EST: Expressed sequence tag cDNA; I-Smads: Inhibitory smads; MAPK: Map kinase; MH: MAD homology domains in Smad proteins; PXSP: Proline-any-serine-proline peptide consensus; qPCR: Real-time quantitative RT-PCR; R-Smads: Receptor-regulated smads; RT-PCR: Reverse-transcriptase polymerase chain reaction; TGFβ: Transforming growth factor-β.

## Competing interests

The authors declare that they have no competing interests pertaining to the work and conclusions submitted herein.

## Authors’ contributions

GMS carried out the molecular cloning and sequence alignment, performed the microinjections, western blots, and RT-PCR analyses, photographed the specimens, prepared figures, and drafted the manuscript. WQG guided the sequence alignment and RT-PCR analyses, performed initial microinjections, provided essential experimental and intellectual guidance, and edited the manuscript. JOH designed and cloned the chimera constructs, prepared additional figures, and edited the manuscript. GHT provided fundamental experimental and intellectual guidance, provided materials and clones, and edited the manuscript. All authors read and approved the final manuscript.

## Supplementary Material

Additional file 1 **Protein sequence alignments of R-Smad orthologs.** The alignment highlights functionally and structurally important residues and regions present in R-Smads. In the MH1 domain (orange), there is a nuclear localization signal (brick red), a DNA binding β hairpin (teal), and residues that make up some or most of the hydrophobic core of the molecule (purple) [46]. Four residues coordinate a zinc atom at the center of the molecule (green triangles) [49]. In the MH2 domain (pink), there are sites of trimer stabilization (lilac), residues that are critical for trimerization contacts (green stars), residues that contribute to a hydrophobic pocket (blue stars) to bind a cysteine from an adjacent Smad molecule (open blue star), a ‘loop strand pocket’ involved with macromolecular interactions (moss green), and two C-terminal serines which are phosphorylated to activate the R-Smad (yellow diamonds) [45]. The loop strand pocket of the MH2 region also contains several residues that bestow receptor specificity [42]. Blue and red boxes indicate residues that are sub-type specific between R-Smads [50]. Important residues in the linker region have already been discussed in detail in Figure 1. Note that this is not meant to be a comprehensive list of R-Smad proteins across phyla or of all residues contributing to R-Smad structure or function; please consult references for studies of these and other proteins and functional sites. Click here for file

Additional file 2 **Table of accession numbers and details about proteins used in the alignments.** Details of the orthologs of R-Smads from human, *Xenopus laevis*, *Drosophila melanogaster*, and *Nematostella vectensis* used in this analysis. Click here for file

Additional file 3 **Primer sequences and experimental PCR design to create the chimeric constructs.** The table contains all primers to create all sections of each of the three chimeric constructs. The diagram shows the primers used to amplify particular sections of the constructs. Full constructs were amplified from combined sections by PCR with end-point primers. Relative lengths of the constructs are depicted. See Methods sections for a full explanation of design and method. Click here for file

Additional file 4 **Loading controls for western blots.** Protein translation levels were detected with an antibody to the HA tags of the HA-RSmads expressed from mRNA made in vitro from the pCS2 expression vector. (A) From left to right: protein ladder, XSmad1, NvSmad1, and uninjected control embryos. The non-specific band signals indicate equal protein loading on the gel (blue arrow). (B) Left to right: protein ladder, XSmad2, XSmad3, dSmad2, NvSmad2/3, and uninjected control. 40 kDa β-Actin loading control band can be seen where indicated (blue arrow). (C) Left to right: protein ladder, water injection (control), uninjected embryo, XSmad2, XSmad3, NvSmad2/3, MH1 chimera, MH2 chimera, and linker chimera. Non-specific bands indicate equal loading across the gel (blue arrow). Click here for file

Additional file 5 **Table of RT-PCR primers used on the Roche 480 Light Cycler system.** All of the primers used in our animal cap gene induction assays are provided, with published conditions and references. Click here for file

Additional file 6 **Further examples of Smad2/3 overexpression ‘perturbed axis’ phenotypes.** Examples of the ‘perturbed axis’ phenotype in tadpoles at stages 33 to 34. This phenotype was observed at some level by any of the treatments in our experiments. Click here for file

Additional file 7 **Dosage experiments with three concentrations of XSmad2 and NvSmad2/3.** Dosage experiments showed that increasing or decreasing the mRNA concentration does not significantly change the gene induction patterns produced by XSmad2 and NvSmad2/3 in animal cap assays. Click here for file
