# Supplementary material for: Conservation and evolutionary divergence in the activity of receptor-regulated smads
Source: EvoDevo. 2012 Oct 1;3:22. doi: 10.1186/2041-9139-3-22 (PMC3500652; doi:10.1186/2041-9139-3-22)
Supplement: Additional file 1 — Protein sequence alignments of R-Smad orthologs. The alignment highlights functionally and structurally important residues and regions present in R-Smads. In the MH1 domain (orange), there is a nuclear localization signal (brick red), a DNA binding β hairpin (teal), and residues that make up some or most of the hydrophobic core of the molecule (purple) [46]. Four residues coordinate a zinc atom at the center of the molecule (green triangles) [49]. In the MH2 domain (pink), there are sites of trimer stabilization (lilac), residues that are critical for trimerization contacts (green stars), residues that contribute to a hydrophobic pocket (blue stars) to bind a cysteine from an adjacent Smad molecule (open blue star), a ‘loop strand pocket’ involved with macromolecular interactions (moss green), and two C-terminal serines which are phosphorylated to activate the R-Smad (yellow diamonds) [45]. The loop strand pocket of the MH2 region also contains several residues that bestow receptor specificity [42]. Blue and red boxes indicate residues that are sub-type specific between R-Smads [50]. Important residues in the linker region have already been discussed in detail in Figure 1. Note that this is not meant to be a comprehensive list of R-Smad proteins across phyla or of all residues contributing to R-Smad structure or function; please consult references for studies of these and other proteins and functional sites. [file 2041-9139-3-22-S1.pdf]

Additional File 1.


```
HS2    MSSILP----------------FTPPVVKRLLGWKKSAGGSGGAGGGEQNGQEEKWCEKAVKSLVKKLKKTGR-LDELEKAITTQNCNTKCVTIPSTCSEIWGLSTPNTIDQWDTTGLYS [103]
XS2    MSSILP----------------FTPPVVKRLLGWKKSASGTTGAGGGEQNGQEEKWCEKAVKSLVKKLKKTGQ-LDELEKAITTQNCNTKCVTIPSTCSEIWGLSTPNTIDQWDTTGLYS [103]
HS3    MSSILP----------------FTPPIVKRLLGWKK----------GEQNGQEEKWCEKAVKSLVKKLKKTGQ-LDELEKAITTQNVNTKCITIP------------------------- [68]
XS3    MSSILP----------------FTPPIVKRLLGWKK----------GEQNGQEEKWCEKAVKSLVKKLKKSGQ-LDELEKALTTQSISTKCITIP------------------------- [68]
dSmad2 M---LP----------------FTPQVVKRLLALKK---------GNEDNSVEGKWSEKAVKNLVKKIKKNSQ-LEELERAISTQNCQTRCVTVP------------------------- [66]
NvS23  MTSLLP----------------FTPPVVKRLLGWKR---------GGDE---DDKWAEKAVKSLVKKLKKTGG-LEELEKAITNPGVATKCVTIP------------------------- [66]
HS9a   MHSTTPIS----------SLFSFTSPAVKRLLGWKQ----------GDE---EEKWAEKAVDSLVKKLKKKGAMDELERALSCPGQPSKCVTIP------------------------- [72]
HS9b   MHSTTPIS----------SLFSFTSPAVKRLLGWKQ----------GDE---EEKWAEKAVDSLVKKLKKKGAMDELERALSCPGQPSKCVTIP------------------------- [72]
XS8a   MHASTPIS----------SLFTFTSPAVKRLLGWKQ----------GDE---EEKWAEKAVDSLVKKLKKKGAMEELERALSCPGQPSKCVTIP------------------------- [72]
XS8b   MHASTPIS----------SLFSFTSPAVKRLLGWKQ----------GDE---EEKWAEKAVDSLVKKLKKKGAMEELERALSCPGQPSKCVTIP------------------------- [72]
HS1    MNVT--------------SLFSFTSPAVKRLLGWKQ----------GDE---EEKWAEKAVDALVKKLKKKGAMEELEKALSCPGQPSNCVTIP------------------------- [68]
XS1    MNVT--------------SLFSFTSPAVKRLLGWKQ----------GDE---EEKWAEKAVDALVKKLKKKGAMEELEKALSCPGQPSNCVTIP------------------------- [68]
HS5    MTSMA-------------SLFSFTSPAVKRLLGWKQ----------GDE---EEKWAEKAVDALVKKLKKKGAMEELEKALSSPGQPSKCVTIP------------------------- [69]
dMAD   MDTDDVESNTSSAMSTLGSLFSFTSPAVKKLLGWKQ----------GDE---EEKWAEKAVDSLVKKLKRKGAIEELERALSCPGQPSKCVTIP------------------------- [82]
NvS15  MSSMA-------------SLFSFTPPAVKRLLGWKQ----------GDE---EEKWAEKAIESLVKKLKKKGALEDLEKALSNPNQQSKCVTIA------------------------- [69]
```



```
HS2    FSEQTRSLDGRLQVSHRKGLPHVIYCRLWRWPDLHSHHELKAIENCEYAFNLKKDEVCVNPYHYQRVETPVLPPVLVPRHTEILTELPPLDDYTHSIPENTNF----------------- [206]
XS2    FSEQTRSLDGRLQVSHRKGLPHVIYSRLWRWPDLHSHHELKAVENCEYAFNLKKDEVCVNPYHYQRVETPVLPPVLVPRHTEILTELPPLDDYTHSIPENTNF----------------- [206]
HS3    -----RSLDGRLQVSHRKGLPHVIYCRLWRWPDLHSHHELRAMELCEFAFNMKKDEVCVNPYHYQRVETPVLPPVLVPRHTEIPAEFPPLDDYSHSIPENTNF----------------- [166]
XS3    -----RSLDGRLQVSHRKGLPHVIYCRLWRWPDLHSHHELRAMEVCEYAFSMKKDEVCVNPYHYQRVETPVLPPVLVPRNTEIPAEFPSLDDYSHSIPENTNF----------------- [166]
dSmad2 -----RSKPAPAGEHLRKGLPHVIYCRLWRWPDLQSQNELKPLDHCEYAFHLRKEEICINPYHYKKIELSILVPKSLPTPPDSIVDYP-LDNHTHQIPNNTDYNAAIIRSASLSPPQYME [180]
NvS23  -----RSLDGRLQVSHRKGLPHVIYCRLWRWPDLQSHHELRPIEACEFAFSLKKEEVCVNPFHYQRVETPVLPPVLVPRQQSDVPHELPILPEYTR-PENVPF----------------- [163]
HS9a   -----RSLDGRLQVSHRKGLPHVIYCRVWRWPDLQSHHELKPLECCEFPFGSKQKEVCINPYHYRRVETPVLPPVLVPRHSEYNPQLSLLAKFRSASLHSEPLMPHNATYPDSFQQPPCS [187]
HS9b   -----RSLDGRLQVSHRKGLPHVIYCRVWRWPDLQSHHELKPLECCEFPFGSKQKEVCINPYHYRRVETPVLPPVLVPRHSEYNPQLSLLAKFRSASLHSEPLMPHNATYPDSFQQPPCS [187]
XS8a   -----RSLDGRLQVSHRKGLSHVIYCRVWRWPDLQSHHELKPMECCEFPFGSKQKDVCINPYHYRRVETPVLPPVLVPRHSEFNPQLSLLAKFSNTSLNNEPLMPHNATFPESFQQPPRT [187]
XS8b   -----RSLDGRLQVSHRKGLPHVIYCRVWRWPDLQSHHELKPMECCEFPFGSKQKDVCINPYHYRRVETPVLPPVLVPRHSEFNPQLSLLAKFRNTSLNNEPLMPHNATFPESFQQPPCT [187]
HS1    -----RSLDGRLQVSHRKGLPHVIYCRVWRWPDLQSHHELKPLECCEFPFGSKQKEVCINPYHYKRVESPVLPPVLVPRHSEYNPQHSLLAQFRNLGQN-EPHMPLNATFPDSFQQPNSH [182]
XS1    -----RSLDGRLQVSHRKGLPHVIYCRVWRWPDLQSHHELKPLECCEFPFGSKQKEVCINPYHYKRVESPVLPPVLVPRHSEYNPQHSLLAQFRNLEPS-EPHMPHNATFPDSFQQPNSH [182]
HS5    -----RSLDGRLQVSHRKGLPHVIYCRVWRWPDLQSHHELKPLDICEFPFGSKQKEVCINPYHYKRVESPVLPPVLVPRHNEFNPQHSLLVQFRNLSHN-EPHMPQNATFPDSFHQPNNT [183]
dMAD   -----RSLDGRLQVSHRKGLPHVIYCRVWRWPDLQSHHELKPLELCQYPFSAKQKEVCINPYHYKRVESPVLPPVLVPRHSEFAPGHSMLQFNHVAEPSMPHNVSYSNSGFNSHSLSTSN [197]
NvS15  -----RSLDGRLQVSHRKGLPHVIYCRVWRWPDLQSHHELKPLECCEYAFGLKQKEVCINPYHYHRVESPGLPPVLVPRQSEYPRPPPPLPPPFRATDDPPMPYNASFPFTNRQNTSEQS [184]
```

```
HS2    --------------------------------------------------------PAGIEPQSNYIPE--TPPPGYISEDGETSDQQLNQSMDTGSPAE-LSPTTLSPVNHSLDLQPVT [267]
XS2    --------------------------------------------------------PAGIEPQSNYIPE--TPPPGYISEDGETSDQQLNQSMDTGSPAE-LSPSTLSPVNHNLDLQPVT [267]
HS3    --------------------------------------------------------PAGIEPQSN-IPE--TPPPGYLSEDGETSDHQMNHSMDAGSPN--LSPNPMSPAHNNLDLQPVT [225]
XS3    --------------------------------------------------------PAGIEPQINYIPE--TPPPGYLSEDGETSDQ-MNHSIDTGSPN--LSPNSMSPAHSNMDLQPVT [225]
dSmad2 LGGAGPVSVSSSASSTPATAAGGGGGPSSSSSSSSSAASAYQQQQQQLSFGQNMDSQSSVLSVGSSIPNTGTPPPGYMSEDGDPIPDNDNMNMSRLTP-----PA-DA-A-------PVM [286]
NvS23  --------------------------------------------------------PTQEPTNSQYHIQPGTPTD-YISDDACS-DMSGEHSQQNMQIDQQLSPAPDSNNLI--DAQPIQ [223]
HS9a   ALPPSPSHAFSQSPCTASYPHSPGSPSEPESPYQHSV-D-------------------------------TPPLPYHATEASETQSGGQPVDATADRHVVLSIPNG--------DFRPVC [266]
HS9b   ALPPSPSHAFSQSPCTASYPHSPGSPSEPESPYQHS------------------------------------------------------------------------------DFRPVC [229]
XS8a   PFSSSPSNIFPQSPNTVGYPDSPRSSTDPGSPPYQI-TE-------------------------------TPPPPYNAPDLQGNQNRPTADPAECQLVLSALNR---------DFRPVC [265]
XS8b   PFSSSPSNIFSQSPNTVGYPDSPRSSTDPGSPPYQI-TE-------------------------------TPPPPYNAPDLQGNQNRPTADPAECQLVLSALNR---------DFRPVC [265]
HS1    PFPHSPNSSYPNSPGSSSSTYPHSPTSSDPGSPFQMPAD-------------------------------TPPPAYLPPEDPMTQDGSQPMDTNMMAPPLPSEINRG------DVQAVA [264]
XS1    PFPHSPNSSYPNSPGSSST-YPHSPASSDPGSPFQIPAD-------------------------------TPPPAYMPPEDQMTQDNSQPMDTNMMVPNISQD-INRA-----DVQAVA [263]
HS5    PFPLSPNSPYPPSPASST--YPNSPASSGPGSPFQLPAD-------------------------------TPPPAYMPPDDQMGQDNSQPMDTSNNMIPQIMPSISSR-----DVQPVA [264]
dMAD   TSVGSPSSVNSNPNSPYDSLA------------------------------------------------GTPPPAYSPSEDGNSNNPNDGGQLLDAQMGDVAQVS--------------- [254]
NvS15  PSFDMADSGAGYMSEDGGSPRPEPNAMDVDPVN-------------------------------------SPP---SIS-QSAEAMSHVTAVN--------------------------- [236]
```



```
HS2    YSEPAFWCSIAYYELNQRVGETFHASQPSLTVDGFTDPSN--SERFCLGLLSNVNRNATVEMTRRHIGRGVRLYYIGGEVFAECLSDSAIFVQSPNCNQRYGWHPATVCKIPPGCNLKIF [385]
XS2    YSEPAFWCSIAYYELNQRVGETFHASQPSLTVDGFTDPSN--SERFCLGLLSNVNRNATVEMTRRHIGRGVRLYYIGGEVFAECLSDSAIFVQSPNCNQRYGWHPATVCKIPPGCNLKIF [385]
HS3    YCEPAFWCSISYYELNQRVGETFHASQPSMTVDGFTDPSN--SERFCLGLLSNVNRNAAVELTRRHIGRGVRLYYIGGEVFAECLSDSAIFVQSPNCNQRYGWHPATVCKIPPGCNLKIF [343]
XS3    YCEPAFWCSISYYELNQRVGETFHASQPSMTVDGFTDPSN--SERFCLGLLSNVNRNAAVELTRRHIGRGVRLYYIGGEVFAECLSDNAIFVQSPNCNQRYGWHPATVCKIPPGCNLKIF [343]
dSmad2 YHEPAFWCSISYYELNTRVGETFHASQPSITVDGFTDPSN--SERFCLGLLSNINRNEVVEQTRRHIGKGVRLYYIGGEVFAECLSDSSIFVQSPNCNQRYGWHPATVCKIPPGCNLKIF [404]
NvS23  YTEPTYWCSISYYEMNTRVGETFHASQPSLTVDGFTDPSS--SDRFCLGLLSNINRNPPIEMTRKHIGKGVRLYYIGGEVFAECLSDSSIFVQSPNCNQRYNWHPATVCKIPPGCNLKIF [341]
HS9a   YEEPQHWCSVAYYELNNRVGETFQASSRSVLIDGFTDPSNNRN-RFCLGLLSNVNRNSTIENTRRHIGKGVHLYYVGGEVYAECVSDSSIFVQSRNCNYQHGFHPATVCKIPSGCSLKVF [385]
HS9b   YEEPQHWCSVAYYELNNRVGETFQASSRSVLIDGFTDPSNNRN-RFCLGLLSNVNRNSTIENTRRHIGKGVHLYYVGGEVYAECVSDSSIFVQSRNCNYQHGFHPATVCKIPSGCSLKVF [348]
XS8a   YEEPLHWCSVAYYELNNRVGETFQASARSVLIDGFTDPSNNKN-RFCLGLLSNVNRNSTIENTRRHIGKGVHLYYVGGEVYAECVSDSSIFVQSRNCNYQHGFHPSTVCKIPSGCSLKIF [384]
XS8b   YEEPLHWCSVAYYELNNRVGETFQASARSVLIGGFTDPSNNKN-RFCLGLLSNVNRNSTIENTRRHIGKGVHLYYVGGEVYAECVSDSSIFVQSRNCNYQHGFHPSTVCKIPSGCSLKIF [384]
HS1    YEEPKHWCSIVYYELNNRVGEAFHASSTSVLVDGFTDPSNNKN-RFCLGLLSNVNRNSTIENTRRHIGKGVHLYYVGGEVYAECLSDSSIFVQSRNCNYHHGFHPTTVCKIPSGCSLKIF [383]
XS1    YEEPKHWCSIVYYELNNRVGEAFHASSTSVLVDGFTDPSNNRN-RFCLGLLSNVNRNSTIENTRRHIGKGVHLYYVGGEVYAECLSDSSIFVQSRNCNTHHGFHPTTVCKIPSGCSLKIF [382]
HS5    YEEPKHWCSIVYYELNNRVGEAFHASSTSVLVDGFTDPSNNKS-RFCLGLLSNVNRNSTIENTRRHIGKGVHLYYVGGEVYAECLSDSSIFVQSRNCNTHHGFHPTTVCKIPSSCSLKIF [383]
dMAD   YSEPAFWASIAYYELNCRVGEVFHCNNNSVIVDGFTNPSNN-SDRCCLGQLSNVNRNSTIENTRRHIGKGVHLYYVTGEVYAECLSDSAIFVQSRNCNYHHGFHPSTVCKIPPGCSLKIF [373]
NvS15  YQEPLSWCSIAYYELNNRVGELFHAKSTSLIVDGFTDPNTTNSERFCLGLLSNVNRNSTIENTRRHIGKGVHLYYVGGEVYAECLSDSAIFVQSRNCNHSHGFHPTTVCKIPSGCTLKIF [356]
```



```
HS2    NNQEFAALLAQSVNQGFEAVYQLTRMCTIRMSFVKGWGAEYRRQTVTSTPCWIELHLNGPLQWLDKVLTQMGSPSVRCSSMS [467]
XS2    NNQEFAALLAQSVNQGFEAVYQLTRMCTIRMSFVKGWGAEYRRQTVTSTPCWIELHLNGPLQWLDKVLTQMGSPSVRCSSMS [467]
HS3    NNQEFAALLAQSVNQGFEAVYQLTRMCTIRMSFVKGWGAEYRRQTVTSTPCWIELHLNGPLQWLDKVLTQMGSPSIRCSSVS [425]
XS3    NNQEFAALLAQSVNQGFEAVYQLTRMCTIRMSFVKGWGAEYRRQTVTSTPCWIELHLNGPLQWLDKVLTQMGSPSIRCSSVS [425]
dSmad2 NNQEFAALLSQSVSQGFEAVYQLTRMCTIRMSFVKGWGAEYRRQTVTSTPCWIELHLNGPLQWLDRVLTQMGSPRLPCSSMS [486]
NvS23  NNQEFAQLLSQSVNQGFEAVYQLTRMCTIRMSFVKGWGAEYRRQTVTSTPCWIELHLNGPLQWLDKVLTQMGSPAPCSSVS [423]
HS9a   NNQLFAQLLAQSVHHGFEVVYELTKMCTIRMSFVKGWGAEYHRQDVTSTPCWIEIHLHGPLQWLDKVLTQMGSPHNPISSVS [467]
HS9b   NNQLFAQLLAQSVHHGFEVVYELTKMCTIRMSFVKGWGAEYHRQDVTSTPCWIEIHLHGPLQWLDKVLTQMGSPHNPISSVS [430]
XS8a   NNQLFAQLLSQSVNQGFEVVYELTKMCTIRMSFVKGWGAEYHRQDVTSTPCWIEIHLHGPLQWLDKVLTQMGSPHNPISSVS [466]
XS8b   NNQLFAQLLSQSVNQGFEVVYELTKMCTIRMSFVKGWGAEYHRQDVTSTPCWIEIHLHGPLQWLDKVLTQMGSPHNPISSVS [466]
HS1    NNQEFAQLLAQSVNHGFETVYELTKMCTIRMSFVKGWGAEYHRQDVTSTPCWIEIHLHGPLQWLDKVLTQMGSPHNPISSVS [465]
XS1    NNQEFAQLLAQSVNHGFETVYELTKMCTIRMSFVKGWGAEYHRQDVTSTPCWIEIHLHGPLQWLDKVLTQMGSPHNPISSVS [464]
HS5    NNQEFAQLLAQSVNHGFEAVYELTKMCTIRMSFVKGWGAEYHRQDVTSTPCWIEIHLHGPLQWLDKVLTQMGSPLNPISSVS [465]
dMAD   NNQEFAQLLSQSVNNGFEAVYELTKMCTIRMSFVKGWGAEYHRQDVTSTPCWIEIHLHGPLQWLDKVLTQMGSPHNAISSVS [455]
NvS15  NNQEFAQLLSQSVNYGFEAVYELTKMCSIRISFVKGWGAEYHRQDVTSTPCWIEVHLHGPLQWLDKVLSQMGSPRNPISSVS [438]
```
