# Supplementary material for: Conservation and evolutionary divergence in the activity of receptor-regulated smads
Source: EvoDevo. 2012 Oct 1;3:22. doi: 10.1186/2041-9139-3-22 (PMC3500652; doi:10.1186/2041-9139-3-22)
Supplement: Additional file 2 — Table of accession numbers and details about proteins used in the alignments. Details of the orthologs of R-Smads from human, Xenopus laevis, Drosophila melanogaster, and Nematostella vectensis used in this analysis. [file 2041-9139-3-22-S2.pdf]

Additional File 2

Gene	Animal	Protein Accession #	AA Length	MH1	MH2
Smad1	Human	AAH01878.1	465	12-132	265-441
Smad5	Human	AAB95090.1	465	10-133	265-465
Smad9A	Human	NP_001120689.1	467	13-136	267-467
Smad9B	Human	NP_005896.1	430	13-136	230-430
Smad1	*Xenopus*	NP_001079973	464	09-132	264-464
Smad8A	*Xenopus*	AAL86772.1	466	13-136	266-466
Smad8B	*Xenopus*	AAL86773.1	468	13-136	266-466
MAD	*Drosophila*	AAF51142	455	23-146	255-455
Smad1/5	*Nematostella*	ABC88374.1	438	10-133	237-438
Smad2	Human	AAC39657.1	467	56-172	268-443
Smad3	Human	AAL68976.1	425	08-132	224-414
Smad2	*Xenopus*	NP_001084964.1	467	08-172	266-465
Smad3	*Xenopus*	NP_001079320	425	08-132	224-414
SMOX (dSmad2)	*Drosophila*	NP_511079	486	07-130	285-475
"Smad2/3"	*Nematostella*	XP_001631657	423	08-130	222-412
**Xenopus laevis* does not have a Smad5 ortholog					
