# Supplementary figures and images for: Conservation and evolutionary divergence in the activity of receptor-regulated smads
Source: EvoDevo. 2012 Oct 1;3:22. doi: 10.1186/2041-9139-3-22 (PMC3500652; doi:10.1186/2041-9139-3-22)

Additional File 3

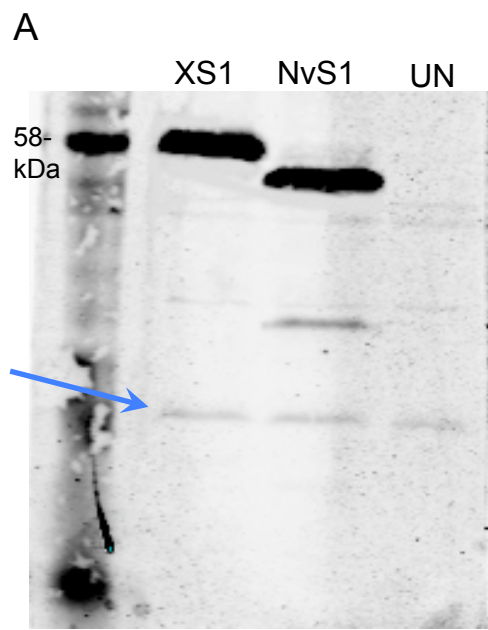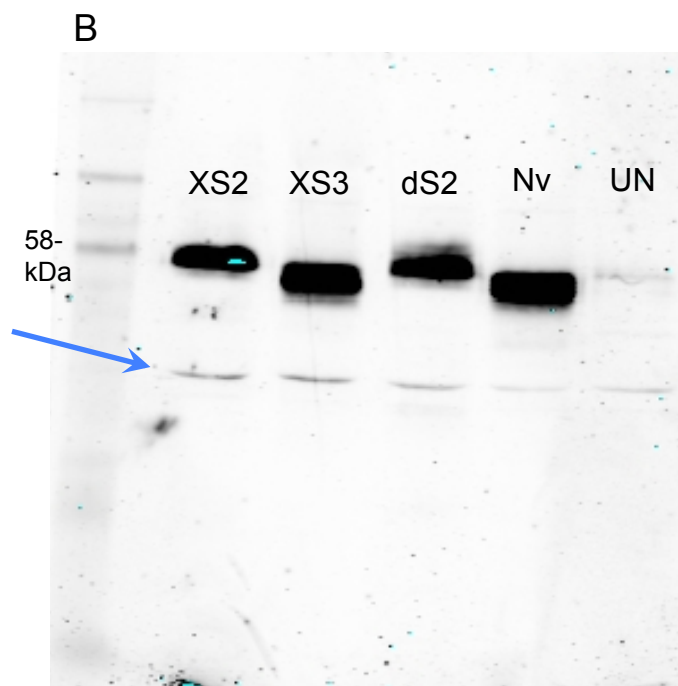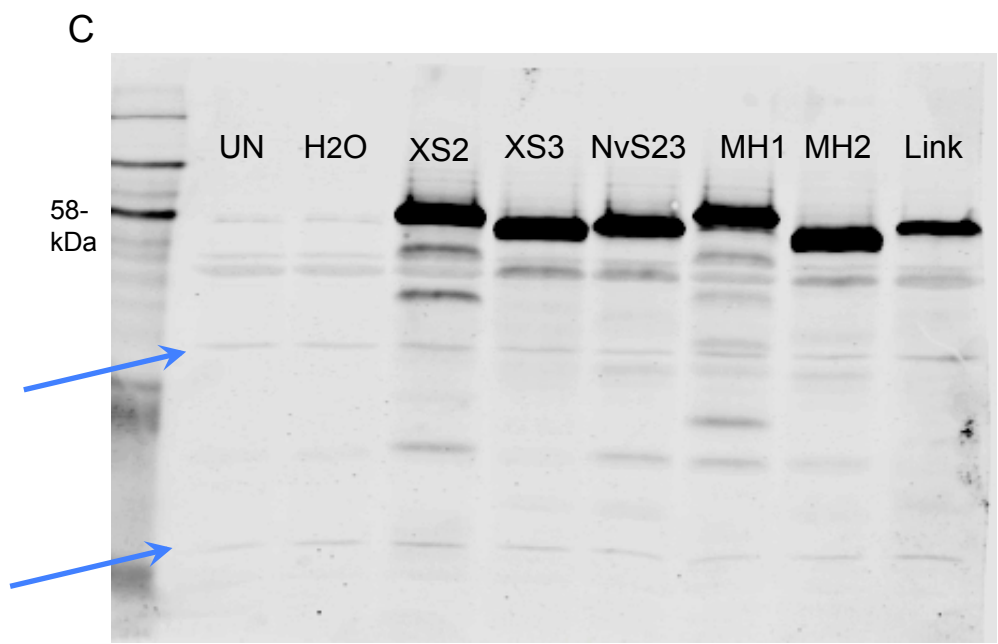

Supplement: Additional file 3 — Primer sequences and experimental PCR design to create the chimeric constructs. The table contains all primers to create all sections of each of the three chimeric constructs. The diagram shows the primers used to amplify particular sections of the constructs. Full constructs were amplified from combined sections by PCR with end-point primers. Relative lengths of the constructs are depicted. See Methods sections for a full explanation of design and method. [file 2041-9139-3-22-S3.pdf]

## Additional File 4

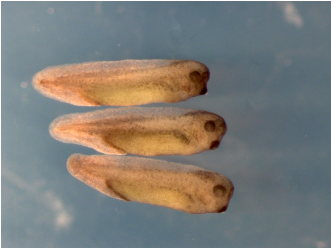

Wild type

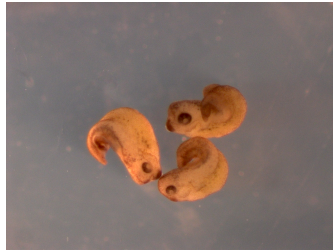

“Perturbed” axis

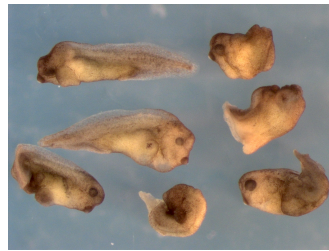

“Perturbed” axis

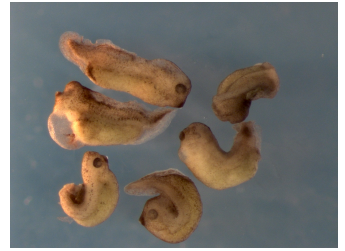

“Perturbed” axis

Supplement: Additional file 4 — Loading controls for western blots. Protein translation levels were detected with an antibody to the HA tags of the HA-RSmads expressed from mRNA made in vitro from the pCS2 expression vector. (A) From left to right: protein ladder, XSmad1, NvSmad1, and uninjected control embryos. The non-specific band signals indicate equal protein loading on the gel (blue arrow). (B) Left to right: protein ladder, XSmad2, XSmad3, dSmad2, NvSmad2/3, and uninjected control. 40 kDa β-Actin loading control band can be seen where indicated (blue arrow). (C) Left to right: protein ladder, water injection (control), uninjected embryo, XSmad2, XSmad3, NvSmad2/3, MH1 chimera, MH2 chimera, and linker chimera. Non-specific bands indicate equal loading across the gel (blue arrow). [file 2041-9139-3-22-S4.pdf]

Additional File 5

Goosecoid

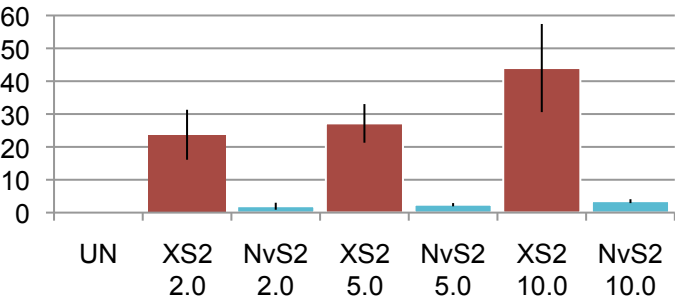

Chordin

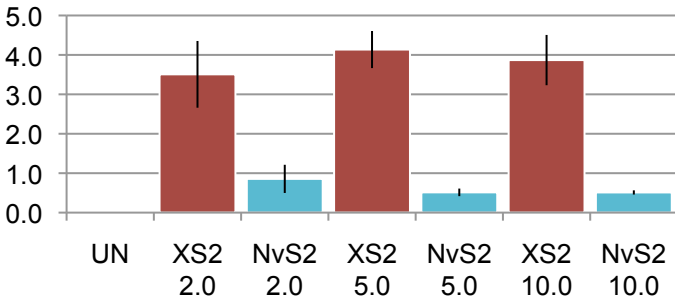

Noggin

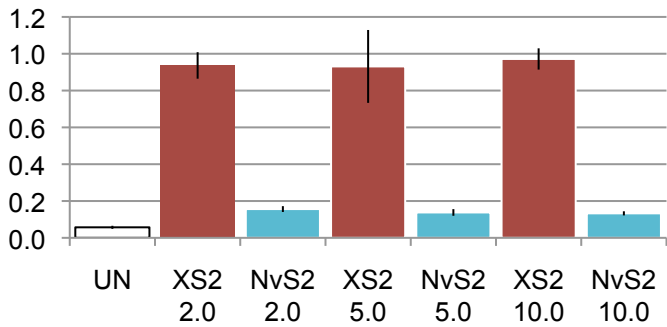

Follistatin

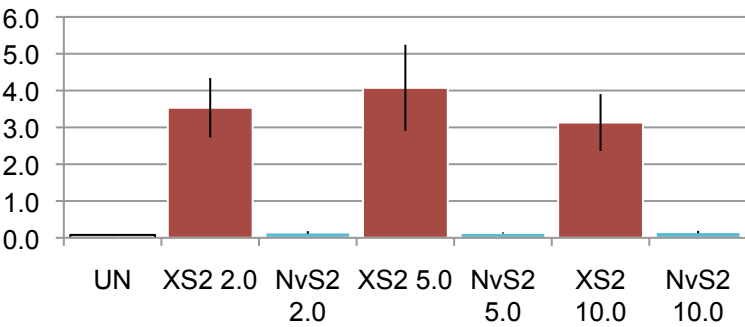

Mixer

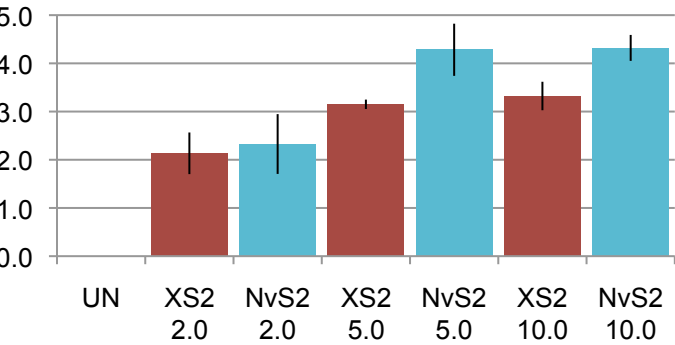

Mix2

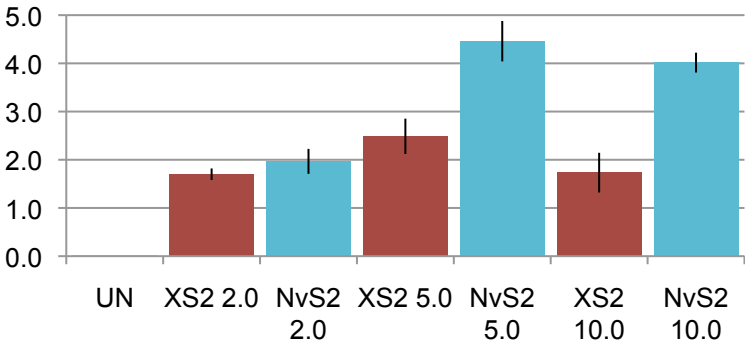

Sox17α

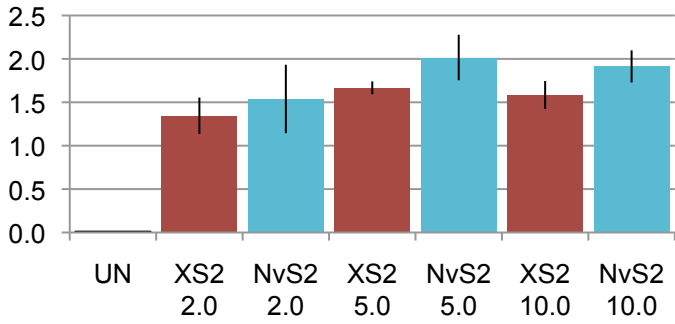

Supplement: Additional file 5 — Table of RT-PCR primers used on the Roche 480 Light Cycler system. All of the primers used in our animal cap gene induction assays are provided, with published conditions and references. [file 2041-9139-3-22-S5.pdf]
