# Supplementary material for: Conservation and evolutionary divergence in the activity of receptor-regulated smads
Source: EvoDevo. 2012 Oct 1;3:22. doi: 10.1186/2041-9139-3-22 (PMC3500652; doi:10.1186/2041-9139-3-22)

Additional File 6

MH1 Chimera

Number	Region		Description	5'-3' Sequence
1	X MH1	F	X start region	ATG TCC TCG ATT TTG CCA TTC CCC CAC CA
2		R	X MH1 end + N linker start	GAA GAA CTG GTG TCT CTA CCC TCT CAT AAT
3	N Linker + MH2	F	X MH1 end + N linker start	GGT AGA GAC ACC AGT TCT TCC ACC AGT GTT
4		R	N end region	TCA CGA GAC AGA GGA GCA GGG GGC AGA GGG

Linker Chimera

Number	Region		Description	5'-3' Sequence
5	N MH1	F	N start region	ATG ACT TCC CTG TTG CCT TTT ACT CCT CCA
6		R	N MH1 end X linker start	GTA AAA CTG GAG TCT CAA CCC GCT GGT AGT
7	X Linker	F	N MH1 end + X linker start	GGT TGA GAC TCC AGT TTT ACC GCC GGT ATT
8		R	X linker end + N MH2 start	TGG CTC TGT ATA GGT GAC AGG TTG CAA ATC
9	N MH2	F	X linker end + N MH2 start	CTG TCA CCT ATA CAG AGC CAA CAT ACT GGT
4		R	N end region	Same as 4

MH2 Chimera

Number	Region		Description	5'-3' Sequence
5	N MH1 + Linker	F	N start region	Same as 5
10		R	N linker end + X MH2 start	AGG TTC CGA ATA CTG TAT AGG CTG TGC ATC
11	X MH2	F	N linker end + X MH2 start	CTA TAC AGT ATT CGG AAC CTG CTT TTT GGT
12		R	X end region	TTA GGA CAT GCT TGA GCA GCG GAC TGA AGG

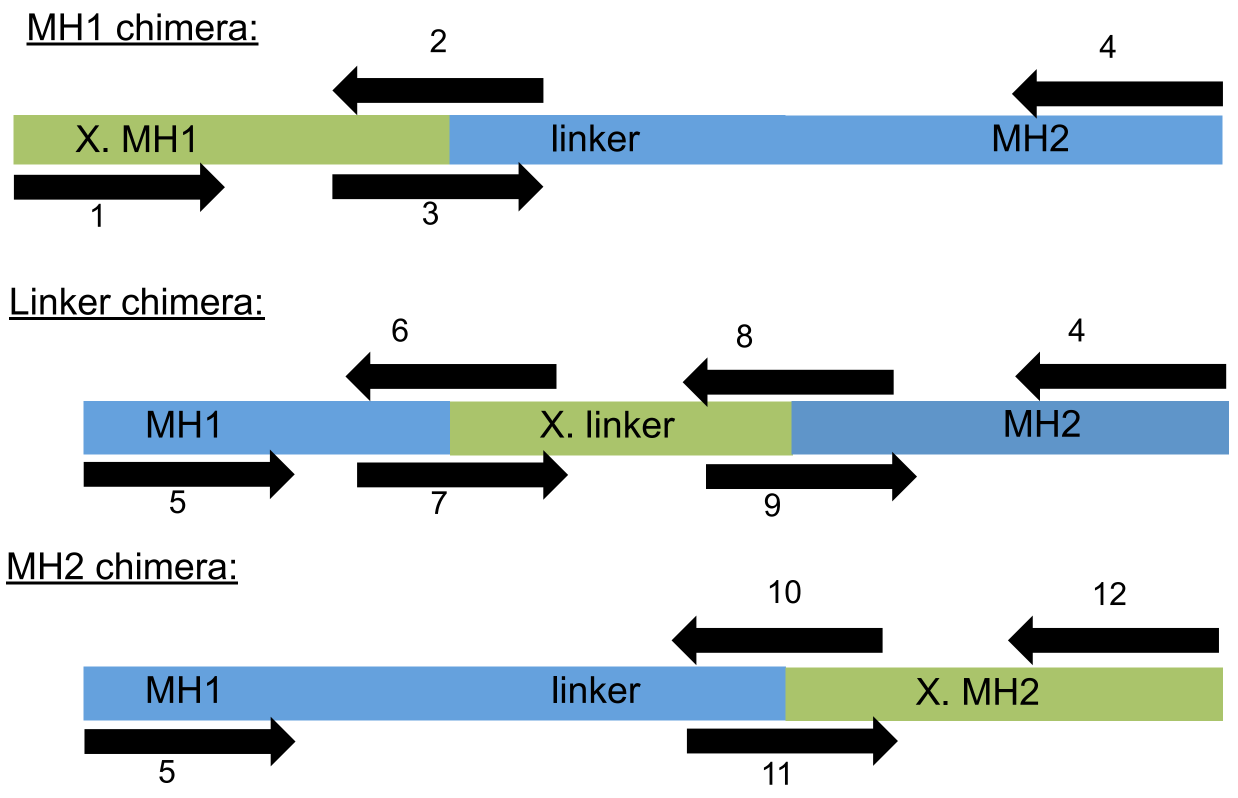

Supplement: Additional file 6 — Further examples of Smad2/3 overexpression ‘perturbed axis’ phenotypes. Examples of the ‘perturbed axis’ phenotype in tadpoles at stages 33 to 34. This phenotype was observed at some level by any of the treatments in our experiments. [file 2041-9139-3-22-S6.pdf]
