# Supplementary material for: Conservation and evolutionary divergence in the activity of receptor-regulated smads
Source: EvoDevo. 2012 Oct 1;3:22. doi: 10.1186/2041-9139-3-22 (PMC3500652; doi:10.1186/2041-9139-3-22)
Supplement: Additional file 7 — Dosage experiments with three concentrations of XSmad2 and NvSmad2/3. Dosage experiments showed that increasing or decreasing the mRNA concentration does not significantly change the gene induction patterns produced by XSmad2 and NvSmad2/3 in animal cap assays. [file 2041-9139-3-22-S7.pdf]

Additional File 7

Gene	Reference	Sequence	Denature	Anneal/ time	Extend/ time	Acquis/ time
Goosecoid	Xanthos et al, 2002	U: 5′-TTC ACC GAT GAA CAA CTG GA-3′	95	55/5	72/11	82/3
		D: 5′-TTC CAC TTT TGG GCA TTT TC –3′				
ADMP	Sander et al, 2007	U: 5'-GAT GAT GGA AGG AGA GGA-3'	95/30	55/60	72/30	
		D: 5'-TCA TGT TCT GAC CCA AAG-3'				
Chordin	XMMR/Xanthos 2002	U: 5'-AAC TGC CAG GAC TGG ATG GT-3'	95	55/5	72/12	81/3
		D: 5'-GGC AGG ATT TAG AGT TGC TTC-3'				
Noggin	XMMR/Xanthos 2002	U: 5'-AGT TGC AGA TGT GGC TCT-3'	95	55/5	72/12	84/3
		D: 5'-AGT CCA AGA GTC TGA GCA-3'				
Follistatin	Agius et al, 2000	U: 5'-CAG TGC AGC GCT GGA AAG AAA T-3'	Unreported			
		D: 5'-TGC GTTGCG GTA ATT CAC TTA C-3'				
Eomesodermin	Thomsen Lab, unpub	U: 5'-TCC TGT CCC CAC AGA CTA ATG-3'	95	55/5	72/12	81/3
		D: 5'-TGA AGG ACT TGA CCT TGA GGA-3'				
Mixer	Henry and Melton 1998	U: 5′-CAC CAG CCC AGC ACT TAA CC-3′	95	55/5	72/12	83/3
		D: 5′-CAA TGT CAC ATC AAC TGA AG-3′				
Mix.2	Xanthos et al, 2001	U: 5'-TGC AAG CCA TCA TTA TTC TAG C-3'	95	55/5	72/11	83/3
		D: 5'-AGG AAC CTC TGC CTC GAG ACA T-3'				
Xsox17	Xanthos et al, 2001	U: 5′-GCA AGA TGC TTG GCA AGT CG-3′	95	58/5	72/8	85/3
		D: 5′-GCT GAA GTT CTC TAG ACA CA-3′				
Xbrachyury	Sun et al, 1999	U: 5′-TTC TGA AGG TGA GCA TGT CG-3′	95	55/5	72/9	75/3
		D: 5′-GTT TGA CTT TGC TAA AAG AGA CAG G-3′				
ODC	Cao et al, 2006	U: 5'-CAA AGC TTG TTC TAC GCA TAG CA-3'	95	55/5	72/5	
		D: 5'-GGT GGC ACC AAA TTT CAC ACT-3'				
Xvent1	Gawantka et al, 1995	U: 5′-GCA TCT CCT TGG CAT ATT TGG-3′	95	62/5	72/20	83/3
		D: 5′-TTC CCT TCA GCA TGG TTC ACC-3′				
Xvent2	Kofron et al, 2001	U: 5'-TGA GAC TTG GGC ACT GTC TG-3'	95	62/5	72/20	83/3
		D: 5'-CCT CTG TTG AAT GGC TTG CT-3'				
Msx1	DeRobertis, unpub	U: 5'-GCT AAA AAT GGC TGC TAA-3'	95	55	72	
		D: 5'-AGG TGG GCT GTG TAA AGT-3'				
Xhox3	Suzuki et al., 1995	U: 5'-ATA TGA TGA GCC ACG CAG CAG-3'	Unreported			
		D: 5'-CAG ATG CTG CAG CTC TTT GGC-3'				

Agius, E. *et al*. 2000. Endodermal Nodal-related signals and mesoderm induction in *Xenopus*. Development 127: 1173-1183.

Cao, Y. *et al*. 2006. *Xenopus* POU factors of subclass V inhibit activin.nodal signaling during gastrulation. Mechanisms of Development 123:614-625

Gawantka, V. *et al*. 1995. Antagonizing the Spemann organizer: role of the homeobox gene *Xvent-1*. The EMBO Journal 14 (24):6268-6279.

Henry, G.L. and D.A. Melton. 1998. *Mixer,* a Homeobox Gene Required for Endoderm Development. Sciec: 281:91-96.

Kofron, M. *et al*. 2001. The Role of Maternal Axin in Patterning the *Xenopus* Embryo. Developmental Biology 237:183-201.

Sander, V. *et al*. 2007. The opposing homeobox genes *Goosecoid* and *Vent1/2* self-regulate *Xenopus* patterning. The EMBO Journal 26:2955-2965.

Sun, B.I. *et al*. 1999. *derriere*: a TGFβ family member required for posterior development in *Xenopus*. Development 126:1467-1482.

Suzuki, A. *et al*. 1995. Bone Morphogenetic Protein acts as a ventral mesoderm modifier in early *Xenopus* embryos. Development, Growth, & Differentiation 37:581-588.

Xanthos, J.B. *et al*. 2002. The roles of three signaling pathways in the formation and function of the Spemann organizer. Development 129:4027-4043.
